# The mutational landscape of ocular marginal zone lymphoma identifies frequent alterations in *TNFAIP3* followed by mutations in *TBL1XR1* and *CREBBP*

**DOI:** 10.18632/oncotarget.14928

**Published:** 2017-02-04

**Authors:** Hyunchul Jung, Hae Yong Yoo, Seung Ho Lee, Sohyun Shin, Sang Cheol Kim, Sejoon Lee, Je-Gun Joung, Jae-Yong Nam, Daeun Ryu, Jae Won Yun, Jung Kyoon Choi, Ambarnil Ghosh, Kyeong Kyu Kim, Seok Jin Kim, Won Seog Kim, Woong-Yang Park, Young Hyeh Ko

**Affiliations:** ^1^ Samsung Genome Institute, Research Institute for Future Medicine, Samsung Medical Center, Seoul, Korea; ^2^ Department of Health Sciences and Technology, Samsung Advanced Institute for Health Sciences and Technology, Sungkyunkwan University, Seoul, Korea; ^3^ Department of Pathology, Samsung Medical Center, Sungkyunkwan University School of Medicine, Seoul, Korea; ^4^ Department of Bio and Brain Engineering, Korea Advanced Institute of Science and Technology (KAIST), Daejeon, Republic of Korea; ^5^ Department of Molecular Cell Biology, Samsung Biomedical Research Institute, Sungkyunkwan University School of Medicine, Suwon, Republic of Korea; ^6^ Division of Hematology and Oncology, Department of Medicine, Samsung Medical Center, Sungkyunkwan University School of Medicine, Seoul, Korea

**Keywords:** marginal zone lymphoma, ocular, whole-genome sequencing, RNA sequencing, mutation

## Abstract

Ocular marginal zone lymphoma is a common type of low-grade B-cell lymphoma. To investigate the genomic changes that occur in ocular marginal zone lymphoma, we analyzed 10 cases of ocular marginal zone lymphoma using whole-genome and RNA sequencing and an additional 38 cases using targeted sequencing. Major genetic alterations affecting genes involved in nuclear factor (NF)-κB pathway activation (60%), chromatin modification and transcriptional regulation (44%), and B-cell differentiation (23%) were identified. In whole-genome sequencing, the 6q23.3 region containing *TNFAIP3* was deleted in 5 samples (50%). In addition, 5 structural variation breakpoints in the first intron of *IL20RA* located in the 6q23.3 region was found in 3 samples (30%). In targeted sequencing, a disruptive mutation of *TNFAIP3* was the most common alteration (54%), followed by mutations of *TBL1XR1* (18%), cAMP response element binding proteins (*CREBBP*) (17%) and *KMT2D* (6%). All *TBL1XR1* mutations were located within the WD40 domain, and *TBL1XR1* mutants transfected into 293T cells increased *TBL1XR1* binding with nuclear receptor corepressor (*NCoR*), leading to increased degradation of *NCoR* and the activation of *NF-κB* and *JUN* target genes. This study confirms genes involving in the activation of the NF-kB signaling pathway is the major driver in the oncogenesis of ocular MZL.

## INTRODUCTION

Extranodal marginal zone lymphoma (EMZL) is a heterogeneous group of low-grade mature B-cell lymphomas that demonstrate different genetic alterations depending on the tumor origin. A number of chromosomal abnormalities have been described in EMZL, including trisomies 3, 12, and 18 and the specific chromosomal translocations t(11;18)(q21;q21), t(1;14)(p22;q32), t(3;14)(p14.1;q32), and t(14;18)(q32;q21). The frequency of chromosomal alterations is dependent on the primary tumor site, which suggests that genetic alterations are associated with different etiologies [1]. t(11;18)(q21;q21), involving the *API2* and *MALT1* genes, is most frequently found in EMZL from the lung (38-53%) and stomach (17-31%) and less commonly in tumors at other sites. t(14;18)(q32;q21), involving the *IGH* and *MALT1* genes, is found in ocular, salivary and cutaneous marginal zone lymphoma (MZL) but not in EMZL from the G-I tract, lung, and thyroid [2]. t(3;14)(p14.1;q32), involving *FOXP1*, has been described in ocular MZL (25%), thyroid MZL (50%), and cutaneous MZL (10%), but has rarely been found in tumors at other sites [3]. t(1;14)(p22;q32) has been reported in intestinal MZL (13%) but not at other sites. Ocular MZL is the second most common type of EMZL and arises from the eyelid, conjunctiva, lacrimal gland and orbit. Overall, 30% of ocular MZL cases involve specific chromosomal translocations for which oncogenic activity is linked to antigen receptor-associated activation of nuclear factor (NF)-κB. In addition to chromosomal translocation, *TNFAIP3*, a negative regulator of the *NF-κB* pathway, was found to be inactivated via somatic deletion and/or mutation in ocular MZL in 12-37% of ocular MZL cases [4]. The fact that these translocations and somatic mutations have been identified in only a minority of ocular MZL cases appears to indicate that additional genomic alterations are involved in the development and progression of ocular MZL. Deep sequencing is currently the method of choice for cataloguing genomic changes in tumors. To obtain a comprehensive overview of the gene expression patterns and genomic alterations in ocular MZL, we produced a multidimensional genomic dataset based on data obtained from whole-genome sequencing (WGS), transcriptome sequencing, and targeted sequencing.

## RESULTS

### Somatic copy number of ocular MZL

**Figure 1:**
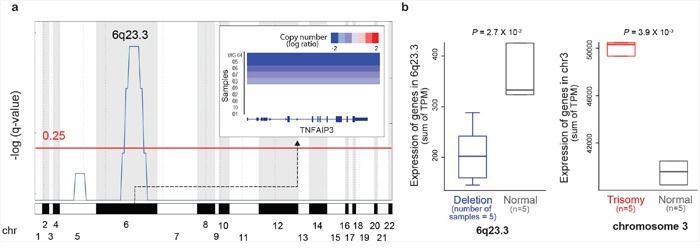
Copy number alterations in ocular MZL **a**. GISTIC analysis of genomic regions with copy number loss.q-values (-log10 transformed) from GISTIC analysis (y-axis) are plotted across the genome (x-axis). Regions with q-values of <0.25 (red line) were considered significant. *TNFAIP3* copy numbers in significantly deleted regions per sample are shown in the top right inset. **b**. The effects of copy number alternations on gene expression. The x-axis denotes samples grouped by copy number status, while the y-axis denotes expression in transcripts per million (TPM). P-values were derived from one-sided t-tests.

To characterize the copy number and structural variations (SVs) of ocular MZL, we performed WGS on 10 matched pairs of tumor and normal samples (Supplementary Table 1). Tumors were sequenced to an average depth of 66x coverage, and matched germline samples were sequenced to 32x coverage (Supplementary Table 2-1). Our somatic copy number variation (CNV) calling [5] identified a total of 14 gain and 63 loss regions (Supplementary Table 3). The gain regions showed broad and low-amplitude changes, whereas the loss regions exhibited narrow and high-amplitude changes (Supplementary Figure 1). One significantly recurrent high-level loss region was identified (Supplementary Figure 2 and Supplementary Table 4). We confirmed the presence of previously identified common genetic alterations in ocular MZL. For example, the 6q23.3 region containing *TNFAIP3*, a negative regulator of the NF-κB signaling pathway, was significantly deleted across tumor samples (Figure 1A; *FDR = 1.02E-03* using GISTIC): 50% of samples (5 of 10) demonstrated the deletion of 6q23.3, where 3 samples had a homozygous deletion (Supplementary Figure 3), and 2 had a heterozygous deletion. RNA-seq data for the same samples (Supplementary Table 2-2) showed a significantly lower average gene expression on 6q23.3 in the deletion samples (n=5) than in samples without the deletion (Figure 1B, *P = 2.70E-02*). We also found trisomies of chromosomes 3 and 18 in 50% (n=5) and 10% (n=1) of samples, respectively (Supplementary Table 3). The trisomy of chromosome 3 increased the mRNA expression of genes on this chromosome: the samples with this amplification (n=5) demonstrated significantly higher gene expression compared with samples without the amplification (Figure 1B).

### Recurrent structural variations associated with the disruption of *TNFAIP3*

**Figure 2:**
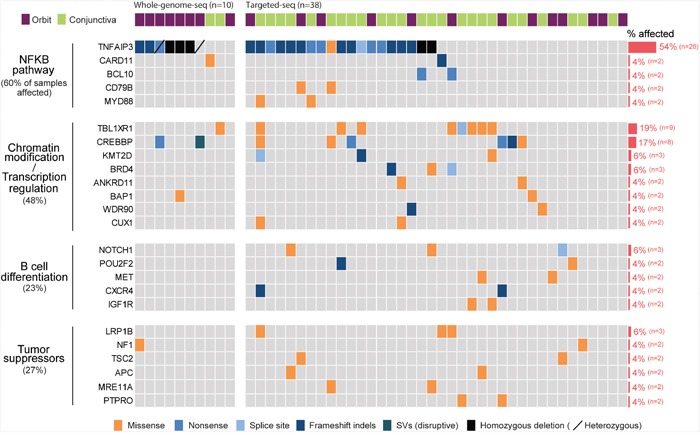
Landscape of somatic alternations in ocular MZL Somatic alternations detected by whole genome (n=10) and targeted sequencing (n=38) are represented as a heatmap. Each column and row represents an affected individual and a gene, respectively. Genes altered in at least two patients were selected, and the fraction of affected individuals per gene is shown on the right. Mutated genes were clustered into four groups according to signaling pathway or molecular function.

We sought to discover five different types of SVs [6] (*i.e*., insertion, deletion, inversion, duplication, and translocation) in each sample (Supplementary Figures 4-5) and identified an average of 40 SVs per sample. Interestingly, we found 5 SV breakpoints in 3 samples (30%) in the first intron of *IL20RA* (Supplementary Figure 6), which encodes a subunit for the interleukin-20 receptor in the 6q23.3 region. The rearrangements included deletions, intra- and inter-chromosomal translocations, and complex SV (Supplementary Figure 7). For example, the WG-06 sample carried multiple rearrangements including 2 inter-chromosomal translocations with different chromosomes and 1 complex SV. As *IL20RA* does not appear to be expressed in lymphoid organs [7], the first intron of *IL20RA* might be a hotspot for structural genomic instability in ocular MZL. In particular, given that *IL20RA* lies upstream (specifically, 1 mb) of *TNFAIP3*, and that all samples with SVs (n=3) carry the homozygous deletion of *TNFAIP3* (Figure 2), these SVs can be thought of as an important mechanism for the complete inactivation of *TNFAIP3*. The known balanced translocations in MALT lymphomas, such as t(11;18)(q21;q21) *API2*-*MALT1*, t(14;18)(q32;q21) *IgH*-*MALT1*, t(1;14)(p22;q32) *BCL10*-*IgH*, and t(3;14) (p14.1;q32) *FOXP1*-*IgH* were not found in our 10 samples. Additionally, no corresponding fusion transcripts were detected in the RNA-seq data.

### Prevalence of the *TNFAIP3* mutation in ocular MZL

We next called somatic single-nucleotide variants (SNVs) and identified an average of 22 non-silent substitutions per tumor sample (range, 12-44; Supplementary Figure 4 and Supplementary Table 5). In total, 70% (n=7) of the samples carried disruptive alterations in *TNFAIP3* (*i.e*., nonsense, frameshift indels, or deletions), and the samples with such alterations were associated with *NF-κB* pathway overexpression (Supplementary Figure 8), which was in line with the notion that *TNFAIP3* inactivation is implicated in the pathogenesis of ocular MZL via the constitutive activation of the *NF-κB* pathway [4]. To obtain a more comprehensive mutational landscape of ocular MZL, we further performed targeted deep sequencing of 430 cancer-related genes in 38 tumor samples (average sequencing depth of 400x per sample; Supplementary Table 2-1). Among a total of 48 tumor samples, 54% (n=26) demonstrated alterations in *TNFAIP3* (Figure 2 and Supplementary Table 5). Our clonality analysis with the whole-genome-sequenced samples revealed that the *TNFAIP3* mutations were all clonal (Supplementary Figure 9 and Supplementary Table 6-1). Together, these results indicated that alterations in T*NFAIP3* are likely early driving events during lymphomagenesis. Other recurrent mutations activating the *NF-κB* pathway included *CARD11* (4%), *BCL10* (4%), *CD79B* (4%), and *MYD88* (4%), all of which are known to function as a positive regulator of *NF-κB* pathway. The majority of the alterations in these genes appear to be activating mutations, including truncating mutations with potential gain-of-function in *BCL10*. Alterations in *BCL10, CARD11*, and *MYD88* have been implicated in ocular MZL pathogenesis [8], and *CD79B* somatic changes in splenic MZL [9] have been reported. These mutations were mutually exclusive to each other while most of them coincided with *TNFAIP3*. A total of 55% of samples demonstrated gene alterations involving activation of the *NF-κB* pathway.

### Frequent alterations in *TBL1XR1*

**Figure 3:**
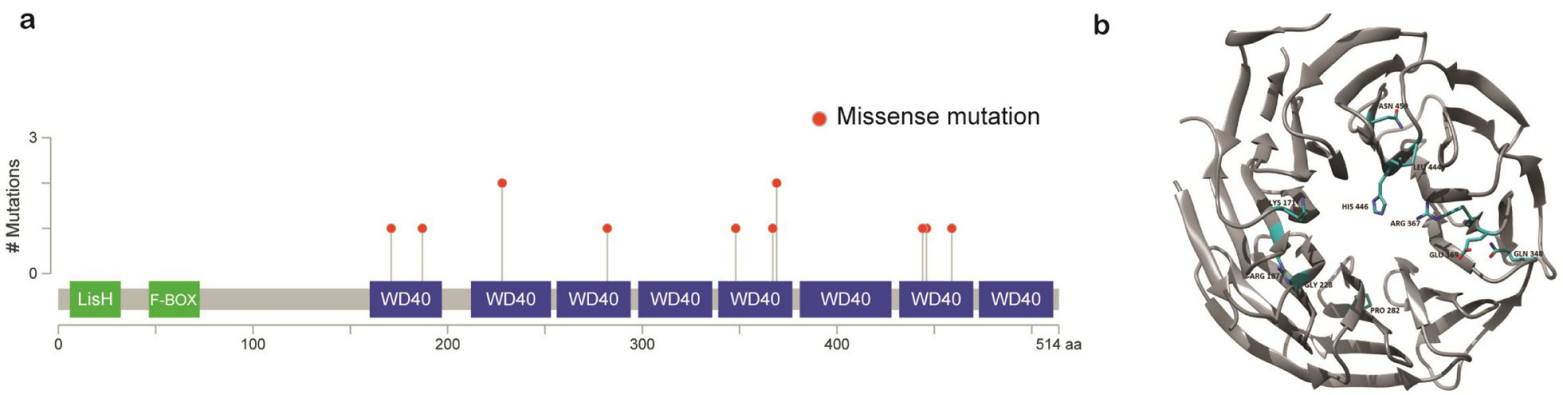
Schematic representation of mutations in *TBL1XR1* **a**. Location of missense mutations in the *TBL1XR1* protein. Frequency of missense mutations (y-axis) is shown by amino acid (x-axis). **b**. Protein structure of *TBL1XR1* with mutations. Mutant residues are highlighted in cyan. Mutations are incorporated into *TBL1XR1* model using swapaa tool of Chimera version 1.10.2 considering appropriate rotamer.

*TBL1XR1*, which is essential for transcriptional repression mediated by unliganded nuclear receptors (NRs) and other regulated transcription factors [10], was the most commonly mutated gene after *TNFAIP3*. Nine out of 48 samples (18%) harbored 13 *TBL1XR1* mutations (Figure 2). The majority of the mutations (92%, n=12) were missense variants, and mutations in all variants were located within the WD40 domain, which has been known to be involved in binding nuclear receptor corepressor (*NCoR*) and histone deacetylase 3 (*HDAC3*) (Figure 3A) [10, 11]. All of these somatic mutations were heterozygotes as validated by Sanger sequencing (Supplementary Figure 10). Since many mutations are located near the top face of the domain and some of them are directly related to the DHSW tetrad, the core scaffold of WD40 domain (Figure 3B), they are expected to affect the binding of *TBL1XR1* to the partner proteins [12]. Mutations in *TBL1XR1* were significantly enriched in conjunctival ocular MZL (*P*=4.63E-02 by χ2 test), suggesting their role in its pathogenesis. Our clonality analysis revealed that the *TBL1XR1* mutations were clonal (Supplementary Figure 9 and Supplementary Table 6-1).

### Alterations of other genes

Genes involved in chromatin modification and/or transcriptional regulation comprised the second most frequently mutated gene group in ocular MZL. cAMP response element binding protein (*CREBBP*), which belongs to the *KAT3* family of histone/protein lysine acetyltransferases [13], was mutated in 17% of samples (n=8). *KMT2D*, which encodes a histone methyltransferase [13], was mutated in 6% of samples (n=3). The majority of the mutations in these genes were truncating variants that include the disruptive SV in *CREBBP*. This is in agreement with previous findings [14] that the loss-of-function of these genes is pathogenically involved in B-cell lymphoma. The other genes mutated in this group include *ANKRD11, BAP1, WDR90*, and *CUX1*. In total, 44% of cases carried mutations in this group, demonstrating that alterations to chromatin modifiers and/or transcription regulators also shape the mutational landscape of ocular MZL. Moreover, genes involved in B-cell differentiation or tumor suppression were also mutated. For example, several genes previously identified in other types of lymphomas, such as *NOTCH1* [15]*, POU2F2* [16]*, CXCR4* [17], and *IGF1R* [18], were affected by mutations. Overall, 23% and 27% of samples harbored mutations in the B-cell differentiation and tumor suppressor groups, respectively. The specific impacts of some of the mutations on lymphomagenesis remain to be investigated.

### Gene expression signature in ocular MZL

**Figure 4:**
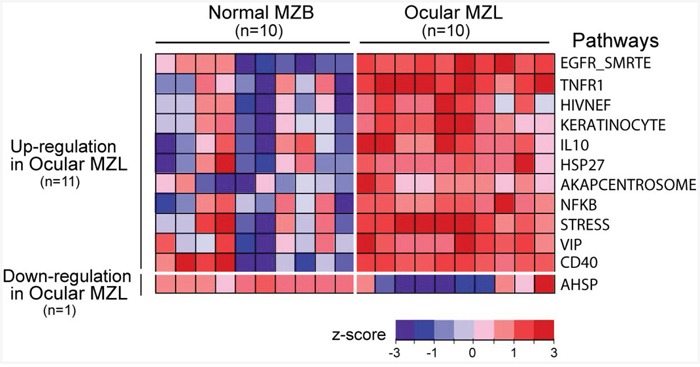
Gene expression signature in ocular MZL Differentially expressed pathways between ocular MZL (n=10) and normal marginal zone B-cell samples (n=10) are shown (p-value <0.01 from a two-sided t-test). The pathway activity level calculated by ssGSEA is a transformed z -score and is shown as a heatmap.

To better delineate the molecular pathogenesis of ocular MZL, we compared the gene expression profiles of ocular MZL samples (n=10) with normal marginal zone B cells (Figure 4; n=10) at the pathway level using single sample Gene Set Enrichment Analysis (ssGSEA). We identified 12 differentially expressed pathways in ocular MZL (p value <0.01), of which 11 were up-regulated, and the other was down-regulated (Supplementary Figure 11). The up-regulated pathways included those playing a crucial role in B-cell proliferation (e.g., *NF-κB, CD40*, and *IL10*) and *NF-κB* crosstalk pathways (e.g., *EGFR_SMRTE, TNFR1*, and *STRESS*), which are known to coordinately activate the *NF-κB* pathway [19, 20], highlighting the fact that aberrant activation of the *NF-κB* pathway is a hallmark of ocular MZL. In addition, apoptosis-suppressing pathways (*HIVNEF, HSP27*, and *VIP*), which confer apoptosis resistance in lymphocytes [21, 22], were over-expressed. The roles of other differentially expressed pathways in ocular MZL development remain to be elucidated. Enriched gene ontology (GO) terms in ocular MZL samples are displayed in Supplementary Figure 12.

### Functional characterization of *TBL1XR1*

To verify the functional characterization of the mutations, we analyzed the interactions of *TBL1XR1* mutants with *NCoR* complex components. These mutations were located in the WD40 domain, which is involved in interactions with *NCoR* and is the part of the corepressor complex that includes *HDAC3*, G-protein pathway suppressor 2 (*GPS2*), silencing mediator of retinoic acid and thyroid hormone receptors (*SMRT*) and *TBL1* [10]. *NCoR* controls the balance between transcriptional repression and activation through ubiquitination-mediated degradation. *HDAC3* is responsible for the deacetylation of histone, which regulates transcriptional repression. *TBL1XR1* is required for transcriptional activation, serving as a corepressor/coactivator exchange factor that regulates the degradation of corepressor components and the recruitment of coactivator complexes [11].

**Figure 5:**
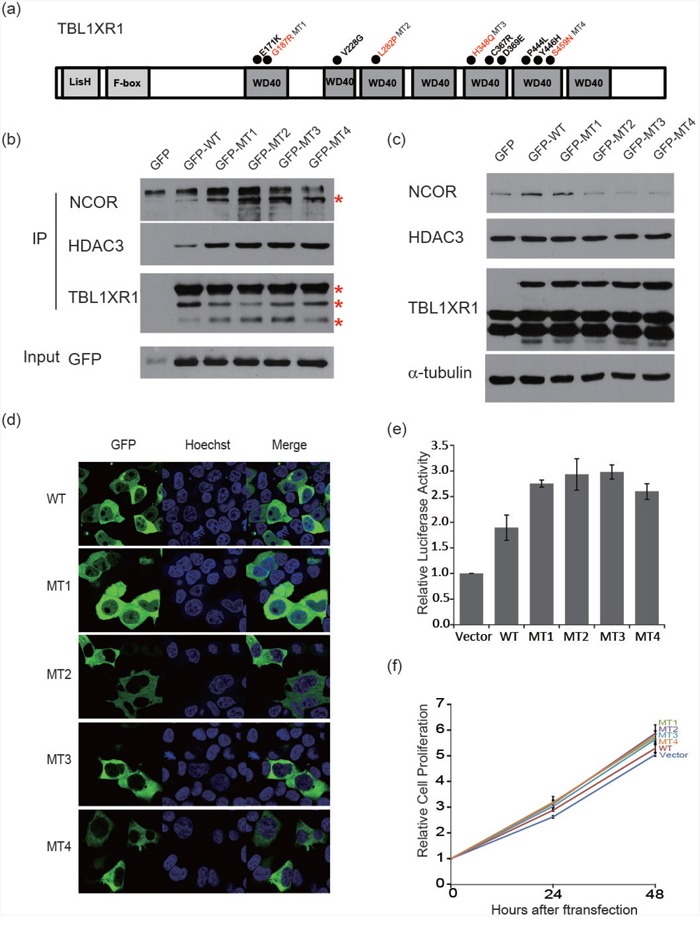
Functional characterization of *TBL1XR1* WD40 domain alternations **a**. Schematic diagram of the structure of *TBL1XR1*. Mutants occurred in the WD40 domain as indicated with circles. **b**. Co-immunoprecipitation of *TBL1XR1* mutants. *TBL1XR1* physically interacts with *NCoR* repressor complexes. Mutant TBL1XR1 interacts with *NCoR* and *HDAC3* more frequently than wild-type *TBL1XR1*. **c**. Expression of *NCoR* protein levels in cells transfected with wild-type or mutant *TBL1XR1*. **d**. Fluorescence images of *TBL1XR1* and Hoechst staining and merged images in 293T cells transfected with each *TBL1XR1* mutant. **e**. *NF-*κ*B* activity assay measuring the effects of each *TBL1XR1* mutant. **f**. Cell proliferation comparison of *TBL1XR1* mutants expressed in cells over 48 h using a CCK-8 assay.

Using four mutants of highly conserved residues (G187R, L282P, H348Q, and S459N), we first analyzed the binding of corepressor complex proteins to *TBL1XR1*. Binding of *NCoR* and *HDAC3* with disease-specific mutants of *TBL1XR1* was increased compared with that of wild-type *TBL1XR1* in a co-immunoprecipitation assay (Figure 5B). Expression of the *NCoR* protein in the total cell lysate was decreased in cells transfected with mutant *TBL1XR1*. The increased binding between *NCoR* and *TBL1XR1* appears to facilitate the degradation of *NCoR*. However, there was no notable difference in *HDAC3* expression levels in mutant *TBL1XR1*-expressing cells (Figure 5C). These results imply that these residues are not directly involved in protein binding, but have a role in maintaining local conformation. The fact that the mutations are not present at the residues in the protein-protein interaction (PPI) hotspot of WD40 domains [12] also supports our interpretation that mutants might induce PPI-enhancing conformational changes rather than abrogate PPI.

Next, we analyzed the localization of wild-type and mutant *TBL1XR1* (Figure 5D). Normally, wild-type *TBL1XR1* is localized in both the nucleus and cytoplasm [23]. When *TBL1XR1* is localized in the nucleus, cell growth is reduced, and apoptosis is increased [24]. *TBL1XR1* mutants transfected into 293T cells demonstrated differential localization compared with the localization of wild-type *TBL1XR1*. Certain *TBL1XR1* mutants (L282P, H348Q, and S459N) tended to localize in the cytoplasm, while another *TBL1XR1* mutant (G187R) was located in both the cytoplasm and nucleus. These changes in the localization of mutant *TBL1XR1* may lead to inhibition of the repressor complex and anti-apoptotic progression [23, 24].

**Figure 6:**
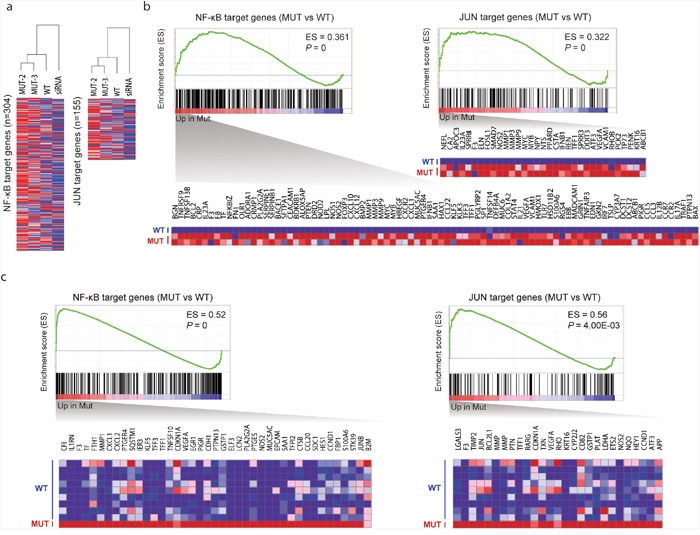
Effects of *TBL1XR1* mutations on gene expression **a**. Hierarchical clustering of gene expression microarray data. An unsupervised hierarchical clustering algorithm was used to group cell lines expressing mutant (L282P and H348Q), wild-type, and siRNA knockdown based on the expression patterns of *NF-κB* (n=304) and *JUN* target genes (n=155). **b**. GSEA of *NF-κB* and *JUN* target gene sets in mutant (n=2; L282P and H348Q) versus wild-type *TBL1XR1* (n=1) based on microarray expression data. Enrichment scores (ES) and p-values are shown in the top right of the enrichment plot. Genes contributing to core enrichment are shown as a heatmap. **c**. GSEA of *NF-κB* and *JUN* target gene sets in mutant (n=1) versus wild-type *TBL1XR1* (n=9) based on RNA-seq data.

The corepressor complex exerts its repressive effects via interactions with transcription factors, including *NF-*κ*B* and *JUN* [10, 25]. Therefore, we examined the extent to which *TBL1XR1* mutations affect the repressive function of this complex, by investigating the expression pattern of *NF-*κ*B* and *JUN* target genes. We first examined gene expression similarities between the samples using microarray expression data for cell lines expressing the mutant (L282P and H348Q) and wild-type *TBL1XR1*, and cell line treated with *TBL1XR1* siRNA (Figure 6A). The mutant samples clustered more closely with the wild-type than the siRNA knockdown sample, suggesting that L282P and H348Q are gain-of-function mutations. We performed gene set enrichment analysis (GSEA) and found that not only the mutant cell lines (Figure 6B) but also a patient sample harboring somatic missense variants (n=2) in *TBL1XR1* (Figure 6C) demonstrated significantly up-regulated *NF-*κ*B* and *JUN* target genes compared with wild-type *TBL1XR1* samples. Based on the *NF-κB* luciferase reporter assay, we verified that expression of the *TBL1XR1* mutants in BJAB (human Burkitt lymphoma) cells enhanced luciferase activity by approximately 1.5-fold compared with that of wild-type *TBL1XR1* (Figure 5E). Overall, these results indicate that *TBL1XR1* mutants can activate the transcription of some transcription factors such *NF-*κ*B* and *JUN* by degrading the corepressor complex. Finally, we analyzed the effects of the *TBL1XR1* mutants on cell proliferation. BJAB cells transfected with constructs expressing the *TBL1XR1* mutants demonstrated a proliferation rate that was approximately 10-15% higher than cells expressing wild-type *TBL1XR1* (Figure 5F).

### Clinical impact of genomic alteration

All samples were thought to be negative for *Chlamydophila psittaci* (*C. psittaci*) based on the absence of WGS reads that aligned *C. psittaci* genome (Online Methods). Mutations in the *MYD88, MET*, and *APC* genes were found in tumor samples from 2 out of 11 patients who relapsed later, and none were found in patients without relapse (0/33) (p=0.054). Otherwise, mutations did not correlate with clinical parameters, including age, sex, international prognostic index, stage, performance status, LDH level, bone marrow involvement, or survival.

## DISCUSSION

Here, we present the mutational landscape of ocular MZL and identify recurrent mutations in multiple genes involving altered signaling pathways. Commonly mutated genes are involved in the NF-kB signaling pathway, B-cell differentiation, and epigenetic modification. These findings are in line with those of previous studies although mutation frequency of each gene was variable according to the study [26, 27].

Ocular MZL arises on the chronic inflammatory background associated with infectious agents and autoimmune disease. Ongoing somatic hypermutation and biased usage of the *IgVH* genes in ocular MZL supports a role for antigen-driven lymphoproliferation and the clonal expansion of lymphoma cells [28]. *C. psittaci* infection is an etiologic agent that has been reported thus far, but this association has demonstrated geographical variability [29]. In our study, no chlamydia sequences were observed with whole-exome sequencing. *NF-κB* is a tightly regulated positive mediator of T- and B-cell development, proliferation, and survival. Constitutive activation of the *NF-κB* pathway plays an essential role in the neoplastic transformation of memory B cells and the progression of MZL [8]. While MZL derived from other sites, particularly the stomach and lung, involves chromosomal translocations of *NF-κB* signaling molecules that provoke antigen-independent MALT lymphoma growth, somatic mutation appears to be the major genetic event that activates *NF-*κ*B* in ocular MZL. Our study confirms alterations in many genes belonging to different functional groups leading to the activation of *NF-*κ*B*, where *TNFAIP3* appears to be the major driver gene in terms of frequency and known functional impact. *TNFAIP3* inhibits *NF-*κ*B* activation by exerting dual ubiquitin-editing functions [30]. *TNFAIP3*-deleted mice with constitutive *NF-*κ*B* activation demonstrate the overproduction of pro-inflammatory cytokines, severe multi-organ inflammation, enhanced proliferation upon activation, and finally develop autoimmune disease [31, 32]. However, *TNFAIP3* inactivation may not be sufficient for the development of MZL and may require additional mutations. The alternative involvement of multiple genes, including *CARD11, BCL10, CD79B, MYD88*, and *TNFRSF14*, converging on the *NF-*κ*B* pathway strongly supports their cooperation in the neoplastic transformation observed in ocular MZL. In addition, MZL involved the somatic mutation of several transcription factors that activate the *NF-*κ*B* pathway. *TBL1XR1*, containing an F-box and the WD40 repeat domains, is a core component of *NCoR* and *SMRT*, which also contain *HDAC3*, transducin (beta)-like 1X (*TBL1X*), and *GPS2* [33]. The F-box-like domain is essential for the recruitment of the 19S proteasome complex to nuclear receptor-regulated transcription units, and WD40 domains are important for binding with proteins such as NCoR [11]. To regulate the switch between corepression/coactivation, *TBL1XR1* functions as an exchange cofactor via the recruitment of the ubiquitin conjugating/19S proteasome to the nuclear receptor corepressors *NCoR* and *SMRT* [34]. *TBL1XR1* is regulated by post transcriptional modification, specifically phosphorylation and sumoylation, and is also required for the activation of the Wnt-β-catenin and *NF-κB* pathways [35, 36].

*TBL1XR1* can become dysregulated by various mechanisms. In solid tumors, dysregulation commonly occurs through gene amplification. *TBL1XR1* overexpression has been observed in diverse cancers [37, 38]. Conversely, hematopoietic neoplasms demonstrate abnormal regulation of *TBL1XR1* through chromosome rearrangement and point mutations [39–41]. Recurrent mutations or deletions have been identified in 19% of primary central nervous system lymphoma cases, Sezary syndrome, and 2/9 ABC-type DLBCLs [39–42]. *TBL1XR1* disrupts glucocorticoid receptor recruitment to the chromatin and results in glucocorticoid resistance in B-ALL [43]. Our study revealed that *TBL1XR1* mutation at the WD40 domain facilitates binding with *NCoR*, leading to increased degradation of the *NCoR* complex, which results in activation of the *NF-κB* and *c-Jun* pathways.

Chromatin modification has emerged as a critical mechanism in lymphomagenesis. As shown here, MZL possesses a broad spectrum of somatic mutations in key genes that are involved in epigenetic regulation. *CREBBP* function as transcriptional coactivators for a large number of DNA binding transcription factors involved in multiple signaling pathways. *CREBBP* is targeted by inactivating mutations and deletions in 39% of DLBCL and 41% of FL cases [44]. *CREBBP* regulates *BCL-2* expression and the rescue of immature B cells from apoptosis [45]. In B-cell lymphoma, mutant *CREBBP* proteins are deficient in acetylating *BCL6* and *p53*, leading to the constitutive activation of *BCL6* and decreased *p53* tumor suppressor activity [46]. Deletion of *CREBBP* down-regulates the transcription of MHC class II genes [47], which contributes to the immune evasion of malignant lymphoma cells. Although the target genes of *CREBBP* mutations have yet to be explored in MZL, inactivating mutations of *CREBBP* may contribute to lymphomagenesis through immune evasion and the regulation of apoptosis. *KMT2D* affects the methylation of lysine 4 on histone H3 (*H3K*4) and the expression of a set of genes, including those involved in the CD40, JAK-STAT, Toll-like receptor and B cell receptor signaling pathways [48]. KMT2D mutations was reported in 22% of ocular MZL in previous study analyzing FFPE samples by targeted deep sequencing [27]. *BRD4* is a BET protein linked to the regulation of *NF-κB* transcriptional activity. *BRD4* maintains nuclear *NF-κB* levels by preventing its ubiquitination and degradation. *BAP1* and *WDR9* are novel chromatin modifiers which associations with malignant lymphoma have not been reported.

Several genes involved in B-cell differentiation were mutated. *CXCR4* is required for the generation of the earliest identifiable B-cell precursor populations in bone marrow and promotes B-cell egress from Peyer's patches [49]. The transcription factor *Oct-2* functions in the terminal phase of B-cell differentiation, and its depletion blocks the *in vivo* differentiation of antibody-secreting plasma cells [50]. The Met/HGF signaling pathway regulates B-cell adhesion in the germinal center and functions in B cell differentiation [51]. Of note, the novel *c-Met* mutation was found only in ocular MZL that later relapsed. Together with *MYD88* and *APC* mutations, the *c-Met* mutation may be a marker for relapse, which is an uncommon event in ocular MZL. Further studies employing a larger cohort will be needed to confirm this hypothesis.

Collectively, the genetic alterations associated with ocular MZL appear to predominantly involve genes that play a role in *NF-*κ*B* signaling pathways, with alterations in genes regulating chromatin modification, transcriptional regulation, and B-cell differentiation. The major driver gene is *TNFAIP3*, which is inactivated by recurrent disruptive structural variations involving the 6q23.3 chromosomal region, along with *TBL1XR1* and *CREBBP*. In addition to these major driver genes, our results expand the directory of mutated genes in ocular MZL, some of which are novel findings. The functions of minor mutated genes and their roles in the initiation and progression of ocular MZL remain to be explored in future studies.

## MATERIALS AND METHODS

### Samples and sequencing

Clinical information for the patients is summarized in Supplementary Table 1. For WGS, 10 tumor samples with matched blood samples were obtained, and for targeted deep sequencing, 38 formalin-fixed, paraffin-embedded (FFPE) MZL tumor tissue samples were utilized. All patient samples were obtained with informed consent at the Samsung Medical Center, Seoul, Korea, and the study was approved by the Institutional Review Board in accordance with the Declaration of Helsinki.

Whole-genome-seq, targeted-seq, and RNA-seq with bioinformatic analysis were performed as described in the Supplementary information. Through the whole exome sequencing of MZL patient samples, potential mutations were selected and validated by Sanger sequencing.

### Functional assays with *TBL1XR1* mutants

#### Cell culture and cell lines

The BJAB (Burkitt lymphoma) cell line and 293T cell line were maintained in RPMI-1640 and DMEM, respectively, containing 10% FBS, 100 U/ml penicillin, 100 μg/ml streptomycin and 250 ng/ml amphotericin B at 37°C and 5% CO_2_. Cell lines were regularly tested for mycoplasma infection using the MycoAlert mycoplasma detection kit (Lonza).

#### Cloning of *TBL1XR1*

A cDNA clone encoding full-length human *TBL1XR1* was isolated from a human placenta cDNA library by PCR amplification. A PCR-generated DNA fragment encoding TBL1XR1 was cloned into the pcDNA3-GFP vector to express proteins with a GFP tag at the N terminus. Mutants of *TBL1XR1*, specifically Gly187Arg (GGA to GTA), Leu282Pro (CTA to CCA), His348Glu (CAT to CAA) and Ser459Asn (AGT to AAT), were generated using the QuikChange kit (Stratagene). Mutant constructs were verified by sequencing.

#### Transfection and RNA interference

To overexpress *TBL1XR1* in the BJAB cell line, plasmids expressing N-terminal GFP- tagged wild-type *TBL1XR1* and *TBL1XR1* mutants (Gly187Arg, Leu282Pro, His348Glu and Ser459Asn) were transfected into cells using the Nucleofector I device (Amaxa) with Nucleofector solution V and the program O-017.

#### Co-immunoprecipitation

For co-immunoprecipitation, whole cell lysates were clarified by centrifugation at 12,000×g for 10 min. Cell lysates were mixed with protein G magnetic beads (Thermo Scientific) and 2 μg of GFP antibody (Abcam) and incubated for 2 h at 4°C on a rotator. Immune complexes were collected using a magnet and washed two times with washing buffer. Whole-cell lysate and beads associated with protein complexes were separated by SDS-PAGE and immunoblotted with appropriate antibodies.

#### Immunoblotting assay

Antibodies used for protein blotting were TBL1XR1 (Novus), HDAC3 and NCoR (Abcam) and alpha-tubulin (Santa Cruz). Horseradish peroxidase (HRP)-conjugated secondary antibodies (Bio-Rad) were used to detect primary antibodies.

#### Immunofluorescence analysis

293T cells were transiently transfected with GFP-tagged *TBL1XR1* expression plasmids using Lipofectamine 3000 transfection reagent (Life Technologies). Then, the nuclei were stained with Nucblue (Hoechst dye, Thermo Scientific). Images were obtained using a confocal laser scanning microscope (CLSM-780, Zeiss).

#### Cell growth assays and NF-κB activity assays

At 48 h after transfection, BJAB cells were seeded into 96-well plates in triplicate at a density of 7.5×10^3^ cells/well with 100 μl of RPMI-1640 containing 10% FBS and antibiotics. Cell growth was analyzed using Cell Counting Kit-8 (Dojindo) according to the manufacturer’s instructions. For the luciferase assay, cells were co-transfected with three *NF-κB* binding site (3xκBL) luciferase vectors and hRL (Renilla luciferase). Then, 5×105 cells were cultured in 24-well plates; after 24 h, the cells were lysed, and firefly and Renilla luciferase activities were measured with a Glomax 20/20 luminometer (Promega) using a dual luciferase reporter assay system (Promega). Firefly luciferase activity was normalized to Renilla luciferase activity.

#### Gene expression analysis

Gene expression analysis using Agilent’s Gene Expression Hybridization Kit (GPL13497) was performed for cell lines expressing the mutant (L282P and H348Q) and wild-type *TBL1XR1*, and cell line treated with *TBL1XR1* siRNA.

## SUPPLEMENTARY DATA TABLES AND FIGURES




