# Correction: The mutational landscape of ocular marginal zone lymphoma identifies frequent alterations in *TNFAIP3* followed by mutations in *TBL1XR1* and *CREBBP*

**DOI:** 10.18632/oncotarget.26080

**Published:** 2018-08-28

**Authors:** Hyunchul Jung, Hae Yong Yoo, Seung Ho Lee, Sohyun Shin, Sang Cheol Kim, Sejoon Lee, Je-Gun Joung, Jae-Yong Nam, Daeun Ryu, Jae Won Yun, Jung Kyoon Choi, Ambarnil Ghosh, Kyeong Kyu Kim, Seok Jin Kim, Won Seog Kim, Woong-Yang Park, Young Hyeh Ko

**Affiliations:** ^1^ Department of Bio and Brain Engineering, Korea Advanced Institute of Science and Technology (KAIST), Daejeon, Republic of Korea; ^2^ Department of Health Sciences and Technology, Samsung Advanced Institute for Health Sciences and Technology, Sungkyunkwan University, Seoul, Korea; ^3^ Department of Pathology, Samsung Medical Center, Sungkyunkwan University School of Medicine, Seoul, Korea; ^4^ Samsung Genome Institute, Research Institute for Future Medicine, Samsung Medical Center, Seoul, Korea; ^5^ Department of Molecular Cell Biology, Samsung Biomedical Research Institute, Sungkyunkwan University School of Medicine, Suwon, Republic of Korea; ^6^ Division of Hematology and Oncology, Department of Medicine, Samsung Medical Center, Sungkyunkwan University School of Medicine, Seoul, Korea

**This article has been corrected:** The correct order of author affiliations is given below:

**Hyunchul Jung**^1,4,*^, Hae Yong Yoo^2,*^, Seung Ho Lee^2,*^, Sohyun Shin^**3**^**, Sang Cheol Kim**^**4**^**, Sejoon Lee**^**4**^**, Je-Gun Joung**^**4**^**, Jae-Yong Nam**^**2,4**^**, Daeun Ryu**^**2,4**^**, Jae Won Yun**^**4,5**^**, Jung Kyoon Choi**^**1**^**, Ambarnil Ghosh**^**5**^**, Kyeong Kyu Kim**^**5**^**, Seok Jin Kim**^**6**^**, Won Seog Kim**^**6**^**, Woong-Yang Park**^**1**^ **and Young Hyeh Ko**^**3**^

^1^ Department of Bio and Brain Engineering, Korea Advanced Institute of Science and Technology (KAIST), Daejeon, Republic of Korea

^2^ Department of Health Sciences and Technology, Samsung Advanced Institute for Health Sciences and Technology, Sungkyunkwan University, Seoul, Korea

^3^ Department of Pathology, Samsung Medical Center, Sungkyunkwan University School of Medicine, Seoul, Korea

^4^ Samsung Genome Institute, Research Institute for Future Medicine, Samsung Medical Center, Seoul, Korea

^5^ Department of Molecular Cell Biology, Samsung Biomedical Research Institute, Sungkyunkwan University School of Medicine, Suwon, Republic of Korea

^6^ Division of Hematology and Oncology, Department of Medicine, Samsung Medical Center, Sungkyunkwan University School of Medicine, Seoul, Korea

* These authors have contributed equally to this work

Original article: Oncotarget. 2017; 8:17038-17049. https://doi.org/10.18632/oncotarget.14928

