# Supplementary material for: The mutational landscape of ocular marginal zone lymphoma identifies frequent alterations in TNFAIP3 followed by mutations in TBL1XR1 and CREBBP
Source: Oncotarget. 2017 Feb 4;8(10):17038–49. doi: 10.18632/oncotarget.14928 (PMC5370020; doi:10.18632/oncotarget.14928)
Supplement: Supplementary file 1 [file oncotarget-08-17038-s001.pdf]

The mutational landscape of ocular marginal zone lymphoma identifies frequent alterations in *TNFAIP3* followed by mutations in *TBL1XR1* and *CREBBP*

SUPPLEMENTARY DATA

DNA and RNA extraction from frozen tumor samples

Tumor DNA (n=10) was extracted using the QIAamp DNA Mini kit (Qiagen, Valencia, CA, USA), according to the manufacturer's protocol. Control DNA from matched peripheral blood samples was extracted with the QIAamp DNA Blood Maxi Kit (Qiagen, Valencia, CA, USA). DNA quality and quantity were analyzed using Nanodrop 8000 UV-Vis spectrometer (NanoDrop Technologies Inc), Qubit ® 2.0 Fluorometer (Life technologies Inc), and 2200 TapeStation Instrument (Aglient Technologies, Santa Clara, CA, USA).

The same frozen tumor samples were used for RNA extraction using a Qiagen RNeasy Mini Kit (Qiagen, Valencia, CA, USA). RNA quality and quantity were assessed by Nanodrop 8000 UV-Vis spectrometer (NanoDrop Technologies Inc. http://www.nanodrop.com) and Agilent 2100 Bioanalyzer Lab-on-a-Chip instrument system (Aglient Technologies, Santa Clara, CA, USA).

DNA extraction from formalin fixation and Paraffin embedding (FFPE) tumor samples

Tumor FFPE tissue DNA was extracted using Maxwell 16 CSC DNA FFPE Kit (Promega Corporation, Madison, USA). DNA quantity and quality were assessed by the same protocol.

Bioinformatics analysis

DNA and RNA data processing

Reads from whole-genome sequencing (WGS) were aligned against the hg19 reference genome using Burrows-Wheeler Aligner (BWA) 0.6.2 [1] and PCR duplicates were marked using Picard (see URLs). The Genome Analysis Toolkit (GATK) [2] was used for the quality score recalibration and local realignment. The same procedure was applied to process reads from Targeted-seq. RSEM [3] was used to align RNA-seq reads against the hg19 reference genome and quantify gene expression level based on UCSC gene model [4]. Picard was used to calculate read alignment statistics. Raw Agilent expression microarray data were preprocessed using GeneSpring GX 13.0 with default options (Agilent Technologies). The natural scale normalized data were further log2 normalized to the control sample (*i.e.,* cell line transfected with empty vector). The normalized probes were collapsed to gene symbols using the CollapseDataset function ("max probe") in GSEA [5].

Somatic variant detection in WGS data

We called somatic single nucleotide variants (SNVs) and indels in WGS data using Strelka [6] and muTect [7] with default settings and then the detected variants were annotated using ANNOVAR [8]. Some of the candidate variants for Sanger sequencing were manually inspected using Integrative Genomics Viewer (IGV) [9]. Structural variations (SVs) were called using Meerkat [10] with default options and somatic SVs were filtered against all normal samples (n=10) to remove polymorphic SVs. Copy number variations (CNVs) were called using BIC-seq2 [11] (bin size: 100; lambda: 1000) and the BIC-seq2 results were used for estimating recurrent CNV regions using GISTIC2.0 [12] with default parameters.

Somatic variant detection in targeted-seq data

We called SNVs and indels using GATK HaplotypeCaller [2] and selected variants with a variant allele frequency (VAF) of 10% and the number of variant supporting reads of 10. We further filtered out variants present in dbSNP 142 [13], 1000 genomes project [14], *ESP* (exome sequencing project) [15], KRGDB (see URLs), or in-house 1000 Korean exome sequencing database. Furthermore, we selected missense variants predicted to have a functional consequence (i.e, damaging or probably damaging) by at least two out of the three methods (SIFT [16], PolyPhen-2 [17], and Mutation Taster [18]). The prediction results were based on ANNOVAR [8] annotation. To identify samples with *TNFAIP3* homozygous deletion, we counted the number of reads aligned on *TNFAIP3* and normalized the count by dividing it by the total number of aligned reads for each sample. Samples with homozygous deletions were defined as those whose normalized read count is less than 2 standard deviations below the mean (Supplementary Table 6-2). Mutations in *TBL1XR1* (Figure 3A) were visualized using MutationMapper on cBioportal [19].

Transcriptome analysis

We obtained canonical pathways from Molecular Signature Database [20] (MSigDB) and used single-sample Gene Set Enrichment analysis [21] (ssGSEA) to infer gene expression-based activity of the pathways. Hierchical Clustering function in GenePattern [22] was utilized to perform clustering of the pathways with default options. FusionMap [23] and deFuse [24] were employed for detection of fusion genes. Normal MZB microarray expression data [25] (IgD+CD27+; n=10) were obtained from ArrayExpress [26] (accession number : E-MTAB-2246) and were combined with tumor RNA-seq data (n=10) using ComBat [27] with default options. GSEA pre-ranked algorithm [5] was run based on differentially expressed genes between *TBL1XR1*-mutant (n=1) and –wild-type samples (n=9) calculated by R package DEGseq [28] with MARS (ranked by z-score). For the microarray expression array data, we used the Diff_of_Classes metric for *TBL1XR1*-mutant (n=2) versus –wild-type samples (n=1) to assign a score and rank the genes. *NF-kB* and *JUN* target genes were obtained from TRRUST database [29].

Clonality analysis

Clonal architecture was inferred based on heterozygous mutations in a region of copy number 2 using SciClone [30]. Mutations were classified as clonal and subclonal if their VAFs are the closest to dominant clone's central VAF and sub-clone's central VAF, respectively. Clones whose central VAF was less than a dominant clone's central VAF were only thought as sub-clones.

URLs

PICARD: http://broadinstitute.github.io/picard/; KRGDB: http://152.99.75.168/KRGDB/menuPages/intro.jsp.

REFERENCES

1. Li H, Durbin R. Fast and accurate short read alignment with Burrows-Wheeler transform. Bioinformatics. 2009; 25: 1754-60. doi: 10.1093/bioinformatics/btp324.
2. McKenna A, Hanna M, Banks E, Sivachenko A, Cibulskis K, Kernytsky A, Garimella K, Altshuler D, Gabriel S, Daly M, DePristo MA. The Genome Analysis Toolkit: a MapReduce framework for analyzing next-generation DNA sequencing data. Genome Res. 2010; 20: 1297-303. doi: 10.1101/gr.107524.110.
3. Li B, Dewey CN. RSEM: accurate transcript quantification from RNA-Seq data with or without a reference genome. BMC Bioinformatics. 2011; 12: 323. doi: 10.1186/1471-2105-12-323.
4. Rosenbloom KR, Armstrong J, Barber GP, Casper J, Clawson H, Diekhans M, Dreszer TR, Fujita PA, Guruvadoo L, Haeussler M, Harte RA, Heitner S, Hickey G, et al. The UCSC Genome Browser database: 2015 update. Nucleic Acids Res. 2015; 43: D670-81. doi: 10.1093/nar/gku1177.
5. Subramanian A, Kuehn H, Gould J, Tamayo P, Mesirov JP. GSEA-P: a desktop application for Gene Set Enrichment Analysis. Bioinformatics. 2007; 23: 3251-3. doi: 10.1093/bioinformatics/btm369.
6. Saunders CT, Wong WS, Swamy S, Becq J, Murray LJ, Cheetham RK. Strelka: accurate somatic small-variant calling from sequenced tumor-normal sample pairs. Bioinformatics. 2012; 28: 1811-7. doi: 10.1093/bioinformatics/bts271.
7. Cibulskis K, Lawrence MS, Carter SL, Sivachenko A, Jaffe D, Sougnez C, Gabriel S, Meyerson M, Lander ES, Getz G. Sensitive detection of somatic point mutations in impure and heterogeneous cancer samples. Nat Biotechnol. 2013; 31: 213-9. doi: 10.1038/nbt.2514.
8. Yang H, Wang K. Genomic variant annotation and prioritization with ANNOVAR and wANNOVAR. Nat Protoc. 2015; 10: 1556-66. doi: 10.1038/nprot.2015.105.
9. Robinson JT, Thorvaldsdottir H, Winckler W, Guttman M, Lander ES, Getz G, Mesirov JP. Integrative genomics viewer. Nat Biotechnol. 2011; 29: 24-6. doi: 10.1038/nbt.1754.
10. Yang L, Luquette LJ, Gehlenborg N, Xi R, Haseley PS, Hsieh CH, Zhang C, Ren X, Protopopov A, Chin L, Kucherlapati R, Lee C, Park PJ. Diverse mechanisms of somatic structural variations in human cancer genomes. Cell. 2013; 153: 919-29. doi: 10.1016/j.cell.2013.04.010.
11. Xi R, Lee S, Xia Y, Kim TM, Park PJ. Copy number analysis of whole-genome data using BIC-seq2 and its application to detection of cancer susceptibility variants. Nucleic Acids Res. 2016. doi: 10.1093/nar/gkw491.
12. Mermel CH, Schumacher SE, Hill B, Meyerson ML, Beroukhim R, Getz G. GISTIC2.0 facilitates sensitive and confident localization of the targets of focal somatic copy-number alteration in human cancers. Genome Biol. 2011; 12: R41. doi: 10.1186/gb-2011-12-4-r41.
13. Sherry ST, Ward MH, Kholodov M, Baker J, Phan L, Smigielski EM, Sirotkin K. dbSNP: the NCBI database of genetic variation. Nucleic Acids Res. 2001; 29: 308-11. doi:
14. Genomes Project C, Abecasis GR, Auton A, Brooks LD, DePristo MA, Durbin RM, Handsaker RE, Kang HM, Marth GT, McVean GA. An integrated map of genetic variation from 1,092 human genomes. Nature. 2012; 491: 56-65. doi: 10.1038/nature11632.
15. Fu W, O'Connor TD, Jun G, Kang HM, Abecasis G, Leal SM, Gabriel S, Rieder MJ, Altshuler D, Shendure J, Nickerson DA, Bamshad MJ, Project NES, et al. Analysis of 6,515 exomes reveals the recent origin of most human protein-coding variants. Nature. 2013; 493: 216-20. doi: 10.1038/nature11690.

16. Ng PC, Henikoff S. SIFT: Predicting amino acid changes that affect protein function. Nucleic Acids Res. 2003; 31: 3812-4.

17. Adzhubei I, Jordan DM, Sunyaev SR. Predicting functional effect of human missense mutations using PolyPhen-2. Curr Protoc Hum Genet. 2013; Chapter 7: Unit7 20. doi: 10.1002/0471142905.hg0720s76.

18. Schwarz JM, Rodelsperger C, Schuelke M, Seelow D. MutationTaster evaluates disease-causing potential of sequence alterations. Nat Methods. 2010; 7: 575-6. doi: 10.1038/nmeth0810-575.

19. Gao J, Aksoy BA, Dogrusoz U, Dresdner G, Gross B, Sumer SO, Sun Y, Jacobsen A, Sinha R, Larsson E, Cerami E, Sander C, Schultz N. Integrative analysis of complex cancer genomics and clinical profiles using the cBioPortal. Sci Signal. 2013; 6: pl1. doi: 10.1126/scisignal.2004088.

20. Liberzon A, Subramanian A, Pinchback R, Thorvaldsdottir H, Tamayo P, Mesirov JP. Molecular signatures database (MSigDB) 3.0. Bioinformatics. 2011; 27: 1739-40. doi: 10.1093/bioinformatics/btr260.

21. Barbie DA, Tamayo P, Boehm JS, Kim SY, Moody SE, Dunn IF, Schinzel AC, Sandy P, Meylan E, Scholl C, Frohling S, Chan EM, Sos ML, et al. Systematic RNA interference reveals that oncogenic KRAS-driven cancers require TBK1. Nature. 2009; 462: 108-12. doi: 10.1038/nature08460.

22. Reich M, Liefeld T, Gould J, Lerner J, Tamayo P, Mesirov JP. GenePattern 2.0. Nat Genet. 2006; 38: 500-1. doi: 10.1038/ng0506-500.

23. Ge H, Liu K, Juan T, Fang F, Newman M, Hoeck W. FusionMap: detecting fusion genes from next-generation sequencing data at base-pair resolution. Bioinformatics. 2011; 27: 1922-8. doi: 10.1093/bioinformatics/btr310.

24. McPherson A, Hormozdiari F, Zayed A, Giuliany R, Ha G, Sun MG, Griffith M, Heravi Moussavi A, Senz J, Melnyk N, Pacheco M, Marra MA, Hirst M, et al. deFuse: an algorithm for gene fusion discovery in tumor RNA-Seq data. PLoS Comput Biol. 2011; 7: e1001138. doi: 10.1371/journal.pcbi.1001138.

25. Descatoire M, Weller S, Irtan S, Sarnacki S, Feuillard J, Storck S, Guiochon-Mantel A, Bouligand J, Morali A, Cohen J, Jacquemin E, Iascone M, Bole-Feysot C, et al. Identification of a human splenic marginal zone B cell precursor with NOTCH2-dependent differentiation properties. J Exp Med. 2014; 211: 987-1000. doi: 10.1084/jem.20132203.

26. Rustici G, Kolesnikov N, Brandizi M, Burdett T, Dylag M, Emam I, Farne A, Hastings E, Ison J, Keays M, Kurbatova N, Malone J, Mani R, et al. ArrayExpress update--trends in database growth and links to data analysis tools. Nucleic Acids Res. 2013; 41: D987-90. doi: 10.1093/nar/gks1174.

27. Johnson WE, Li C, Rabinovic A. Adjusting batch effects in microarray expression data using empirical Bayes methods. Biostatistics. 2007; 8: 118-27. doi: 10.1093/biostatistics/kxj037.

28. Wang L, Feng Z, Wang X, Wang X, Zhang X. DEGseq: an R package for identifying differentially expressed genes from RNA-seq data. Bioinformatics. 2010; 26: 136-8. doi: 10.1093/bioinformatics/btp612.

29. Han H, Shim H, Shin D, Shim JE, Ko Y, Shin J, Kim H, Cho A, Kim E, Lee T, Kim H, Kim K, Yang S, et al. TRRUST: a reference database of human transcriptional regulatory interactions. Sci Rep. 2015; 5: 11432. doi: 10.1038/srep11432.

30. Miller CA, White BS, Dees ND, Griffith M, Welch JS, Griffith OL, Vij R, Tomasson MH, Graubert TA, Walter MJ, Ellis MJ, Schierding W, DiPersio JF, et al. SciClone: inferring clonal architecture and tracking the spatial and temporal patterns of tumor evolution. PLoS Comput Biol. 2014; 10: e1003665. doi: 10.1371/journal.pcbi.1003665.

Supplementary Table 1: Clinical information of patients

See Supplementary File 1

Supplementary Table 2-1: Summary of WGS and Targeted-seq QC

Supplementary Table 2-2: Summary of RNA-seq QC

See Supplementary File 1

Supplementary Table 3: List of identified CNVs

See Supplementary File 1

Supplementary Table 4: Identified significant regions from GISTIC analysis

cytoband	6q23.3
q value	0.0010219
residual q value	0.0010219
wide peak boundaries	chr6:137341112-139809220
genes in wide peak	hsa-mir-3145
	IFNGR1
	TNFAIP3
	CITED2
	HEBP2
	CCDC28A
	HECA
	IL20RA
	KIAA1244
	NHSL1
	C6orf115
	PBOV1
	PERP
	REPS1
	IL22RA2
	OLIG3
	TXLNB
	ECT2L
	FLJ46906
	LOC645434
	MIR3145
	LOC100507462

Supplementary Table 5: List of identified SNVs

See Supplementaty File 1

Supplementary Table 6-1: Clonality analysis

Supplementary Table 6-2: Aligned reads in A20 from Targeted-seq

See Supplementaty File 1

Supplementary Table 7: List of gene-specific PCR primers and oligo sequence

Type	Set. No	Primer Name	Sequence
gDNA PCR primer	Set 1	gDNA 1 F	5'-GCCACAAGAGGAATGACAACC-3'
		gDNA 1 R	5'-CCGAGGGATATGCATCCATACC-3'
		gDNA 1 seq	5'-GGAATGACAACCAAATGGTGAGG-3'
	Set 2	gDNA 2 F	5'-GGCCAGAGCAACCATACTGTG-3'
		gDNA 2 R	5'-CAAGTAGCTACCCAGTCTATACAATG-3'
		gDNA 2 seq	5'-GAGCAACCATACTGTGTGACAC-3'
	Set 3	gDNA 3 F	5'-AACATTACTTGTTAATCATGACCAC-3'
		gDNA 3 R	5'-GGATGTTGATTGGCAGAGCAAC-3'
		gDNA 3 seq	5'-GCAACAACACCTTTGCTTCTTG-3'
	Set4	gDNA 4 F	5'-GGAATGTTTATGTAATTGGCAGC-3'
		gDNA 4 R	5'-GGTAACCTTGCTAGCACCTTAGG-3'
		gDNA 4 seq	5'-GGCAGCTAAGACAAAATACTGC-3'
	Set5	gDNA 5 F	5'-CCCTGCCGTGAGTATGAGGCTC-3'
		gDNA 5 R	5'-GTGAGAACAGCACCAGTGGCTC-3'
		gDNA 5 seq	5'-GTATGAGGCTCTGAGGGTTAGG-3'
	Set6	gDNA 6 F	5'-GGCCACAACTAAGCAACAACAG-3'
		gDNA 6 R	5'-GGCCACAACTAAGCAACAACAG-3'
		gDNA 6 seq	5'-CAACAACAGAAAACCTGAATAATGC-3'
siRNA oligo	si-TBL1XR1	Sense	GAGGUAGAUGUUUGGUACA(dTdT)
		Anti-sense	UGUACCAAACAUCUACCUC(dTdT)

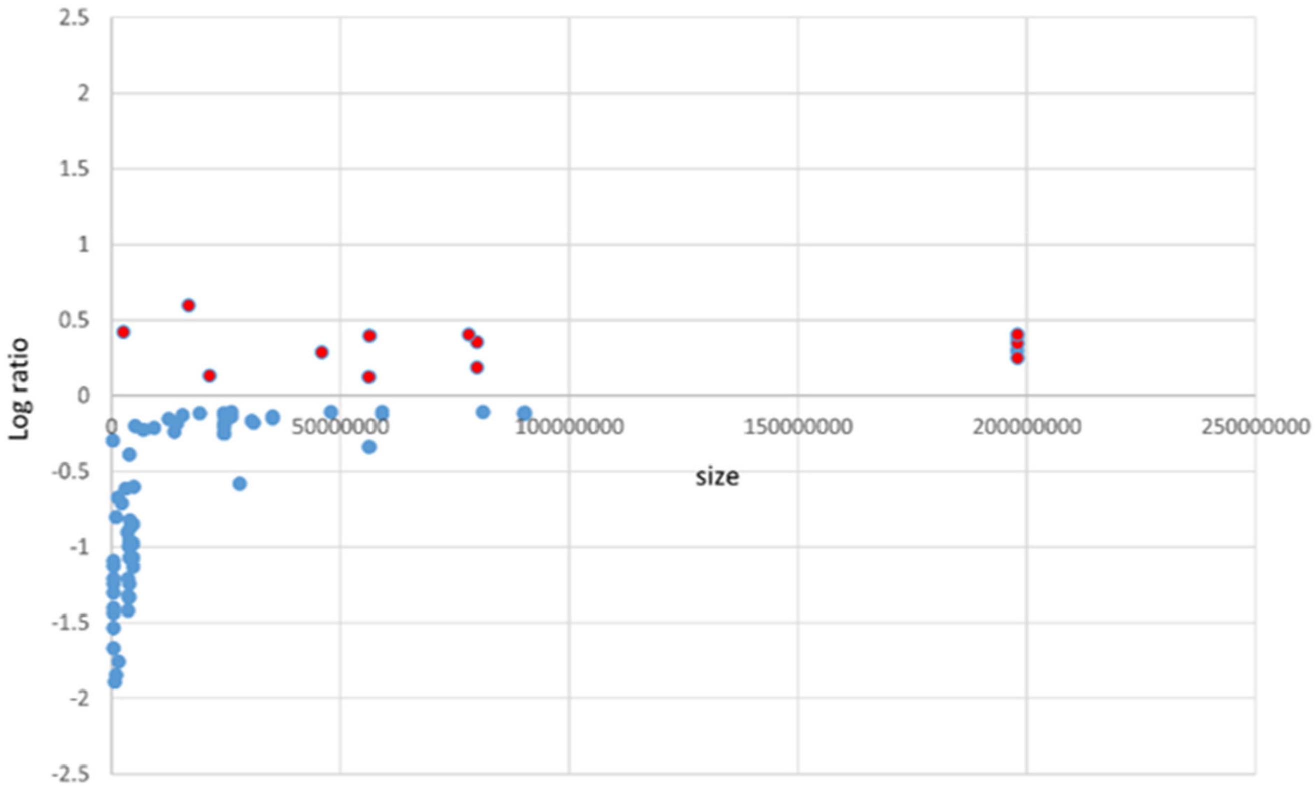

Supplementary Figure 1: Size and amplitude of identified copy number variants. The red circle represents amplification (log ratio > 0.1) while the blue one represent deletion (log ratio < -0.1).

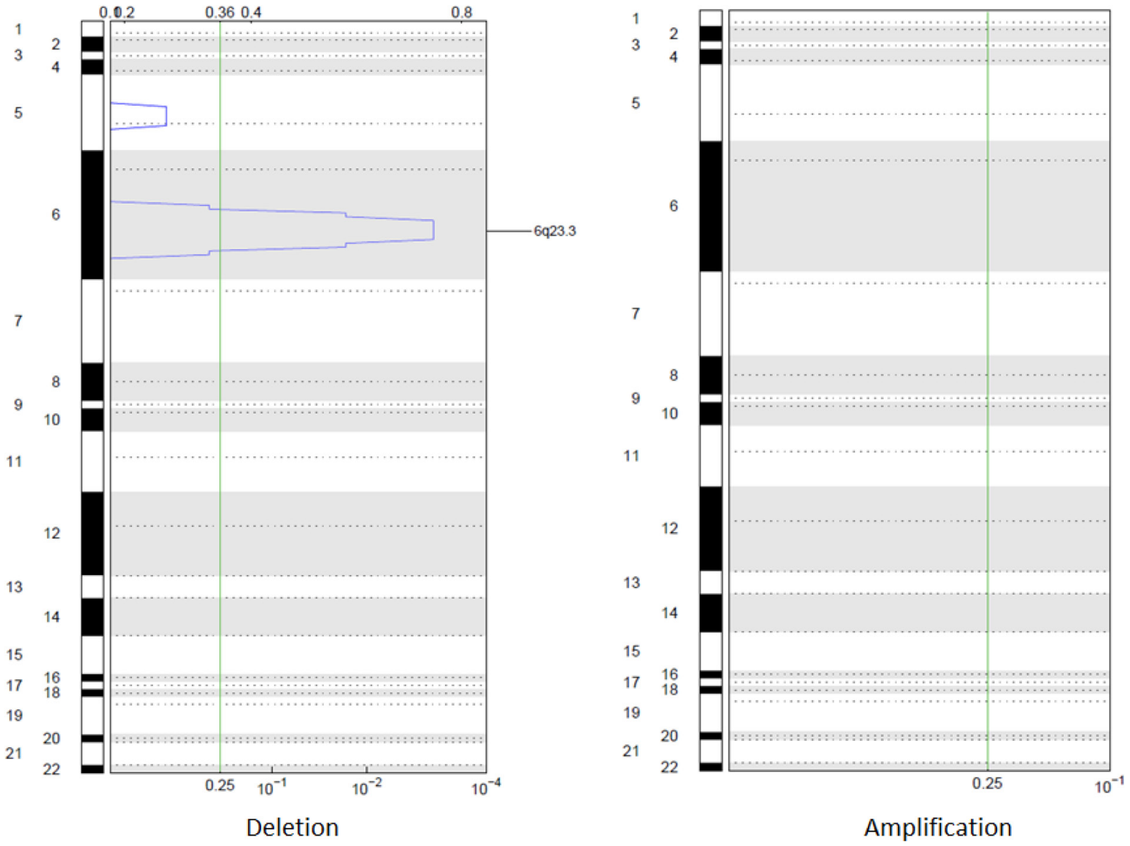

Supplementary Figure 2: Significant peaks of deletion and amplification identified by GISTIC. The green line denotes FDR values < 0.25.

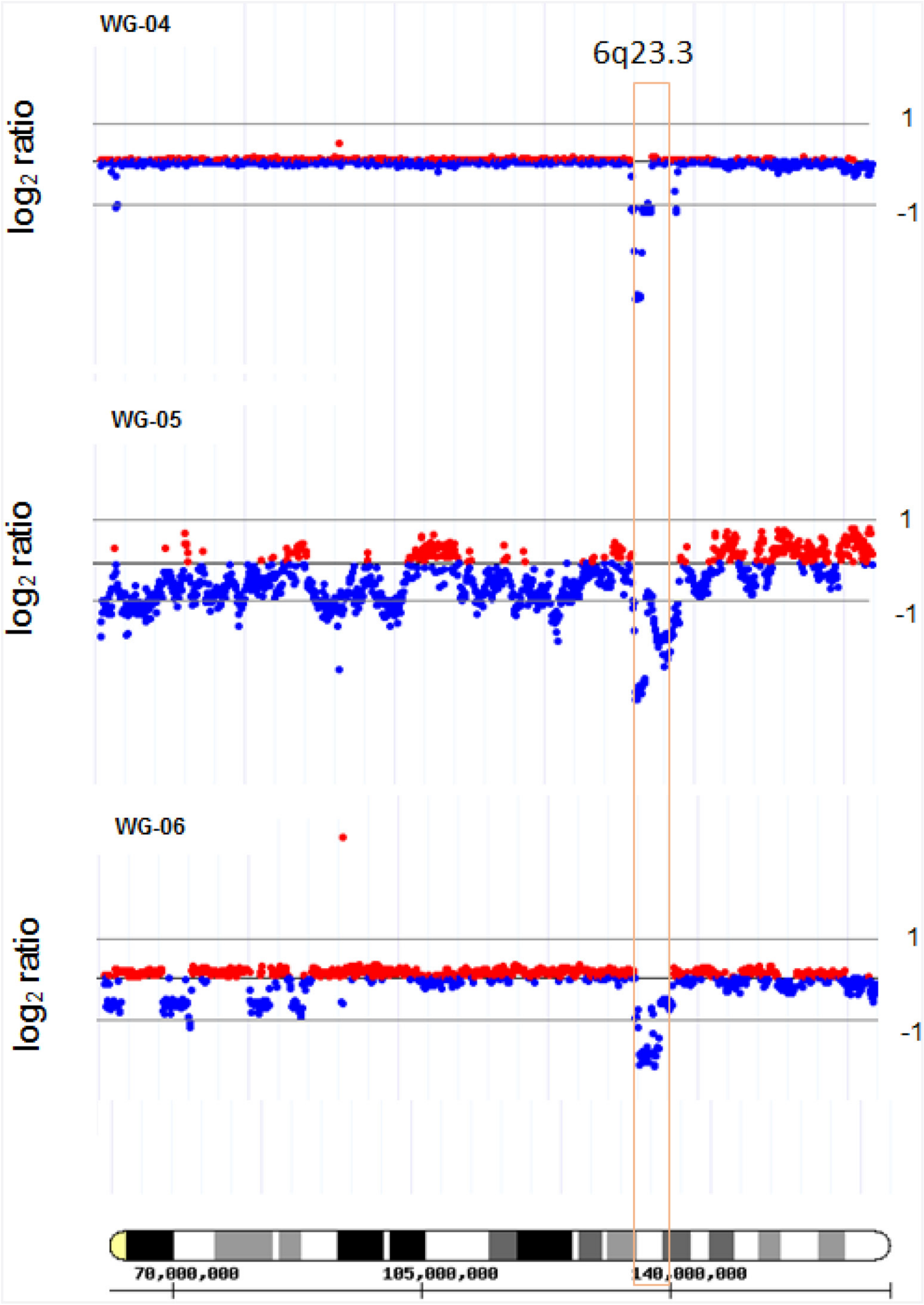

Supplementary Figure 3: Log 2 ratio of copy number for samples (WG-04, -05, and -06) displaying homozygous deletion of *TNFAIP3*. The orange box denotes 6q23.3.

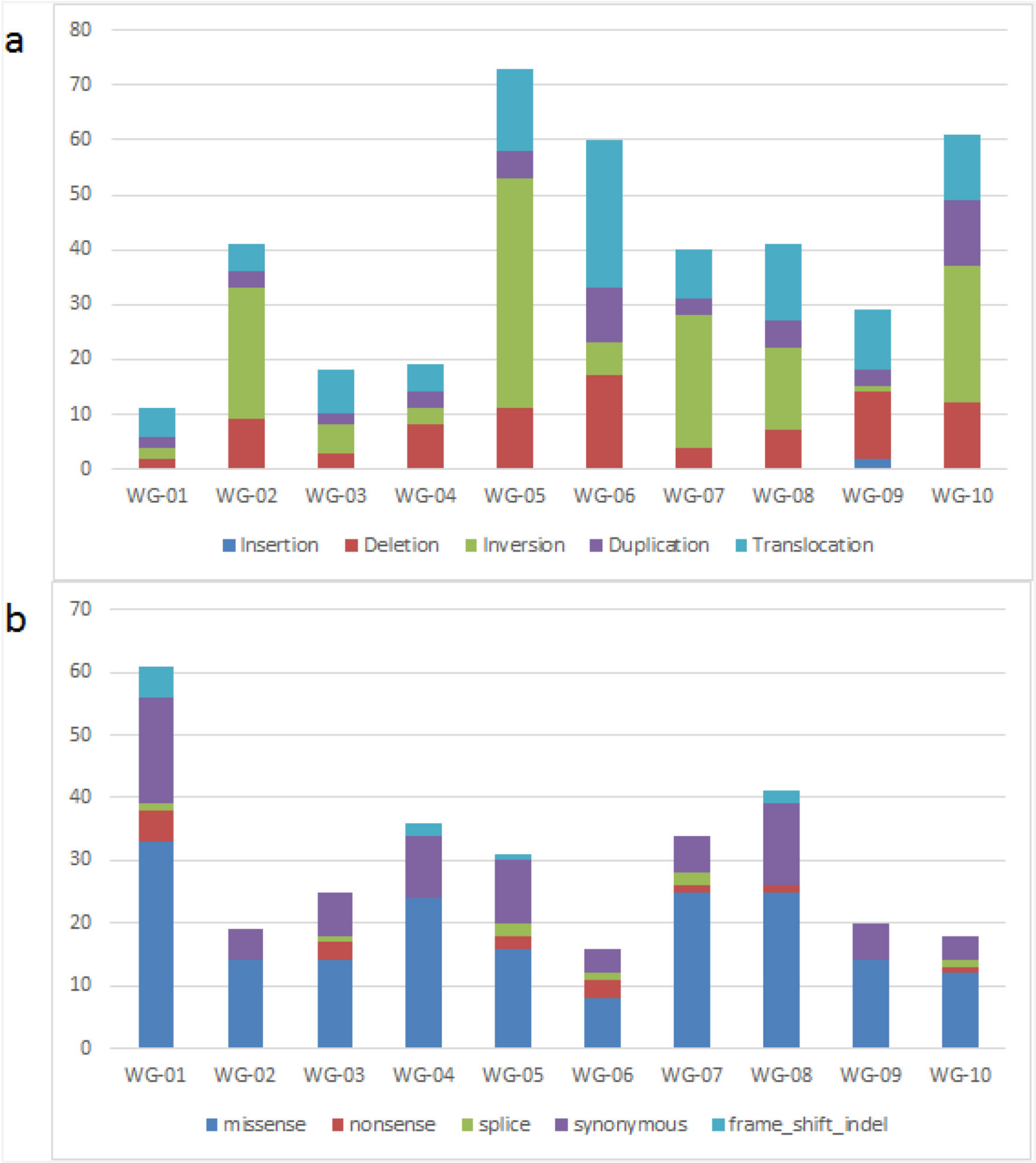

Supplementary Figure 4: Summary statistics of identified somatic structural variations a. and single nucleotide variants b.

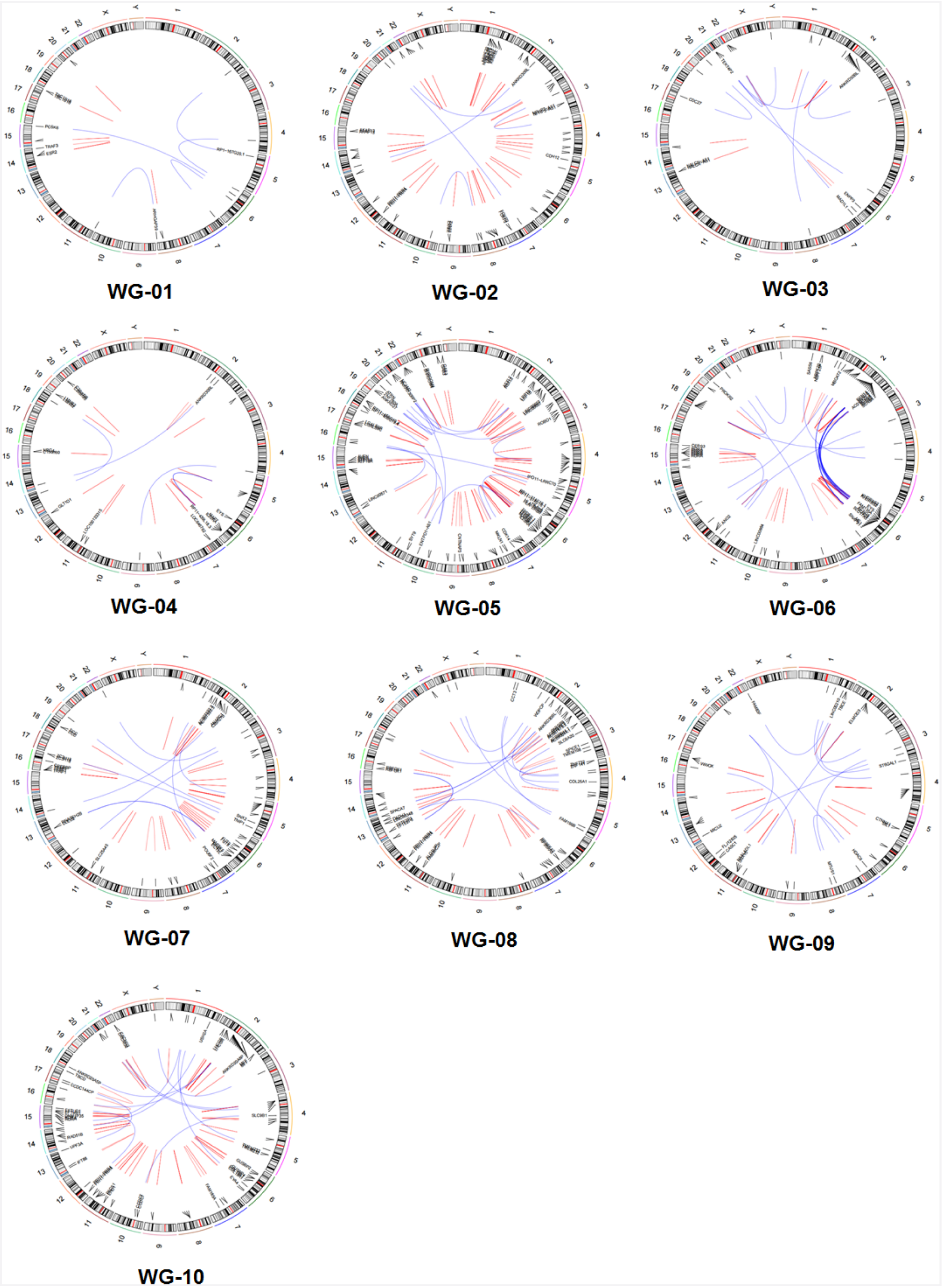

Supplementary Figure 5: Circos plot for the identified structural variations.

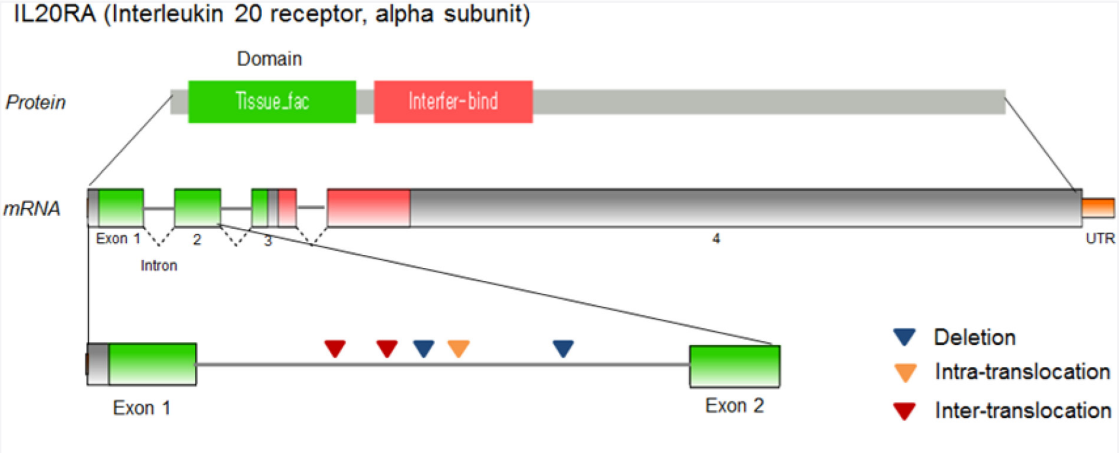

Sample	Breakpoint within IL20RA
WG-04	Deletion
WG-05	Intra-translocation
WG-06	Inter-translocation (2) and Deletion

Supplementary Figure 6: Clustered breakpoints in intron 1 of IL20RA.

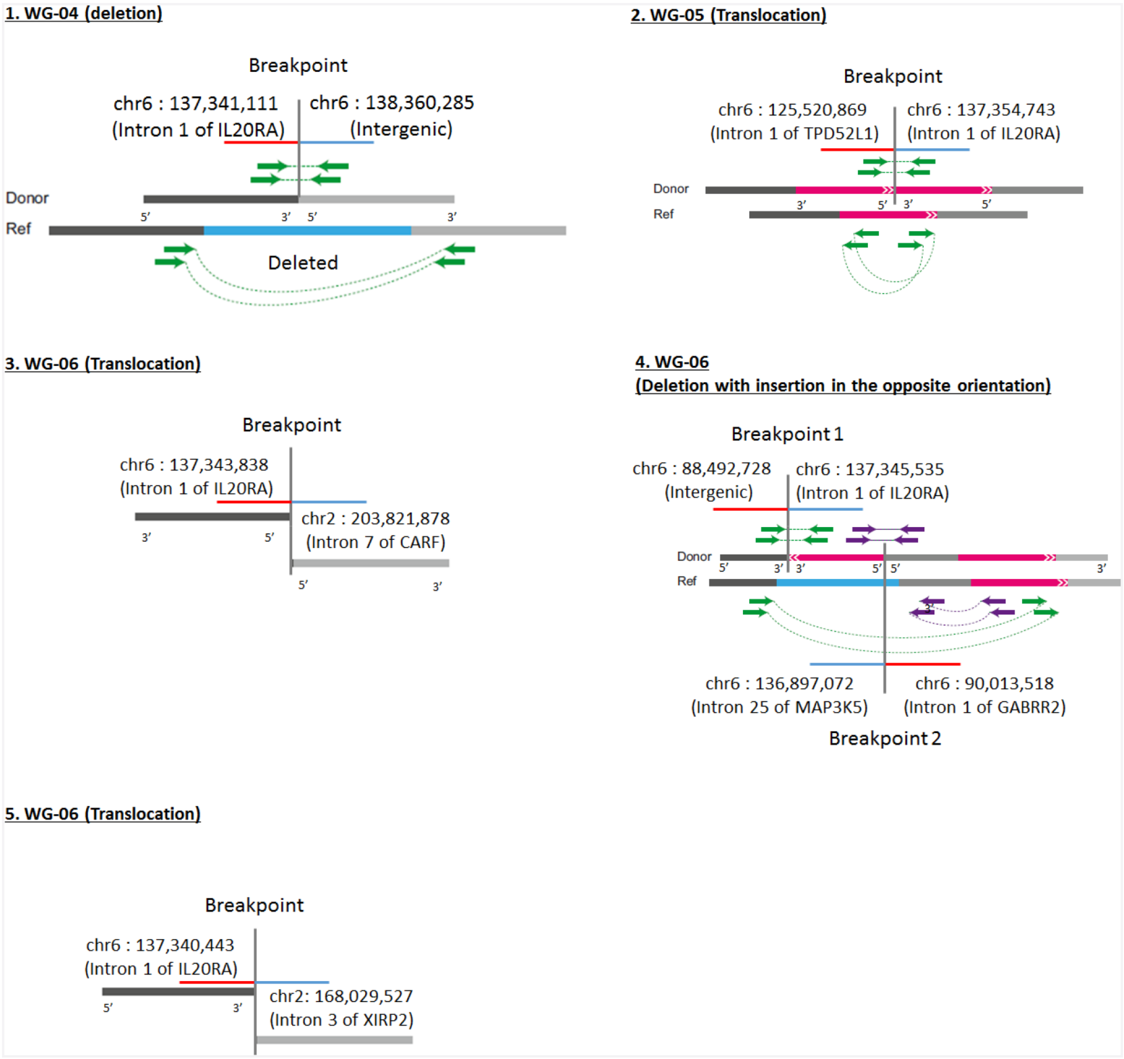

Supplementary Figure 7: Details of breakpoints in intron 1 of IL20RA.

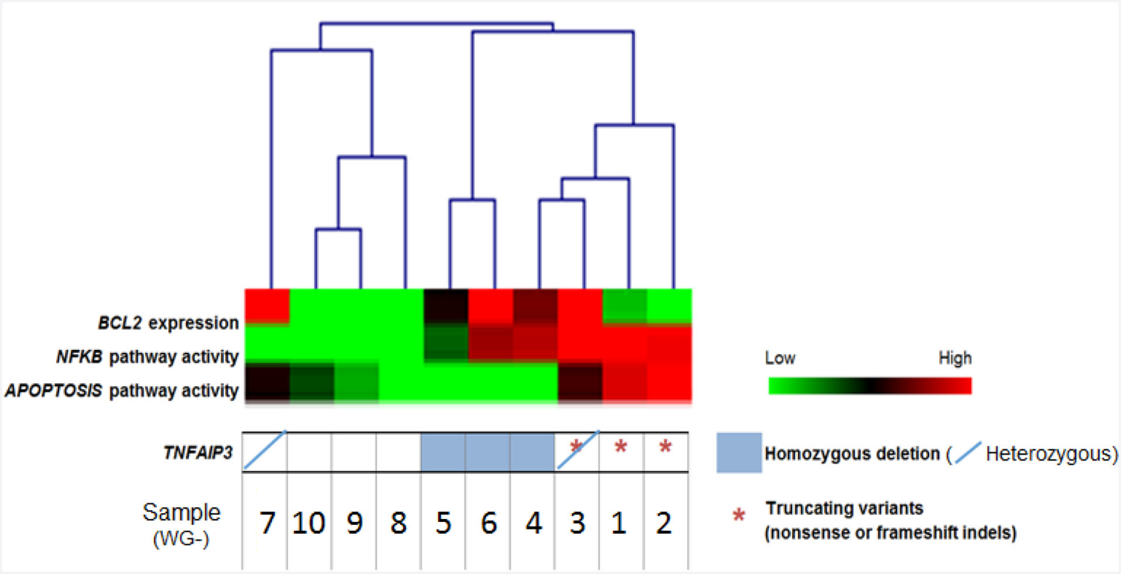

Supplementary Figure 8: Pathway-based hierarchical clustering of expression profiling. ssGSEA was used to calculate enrichment score of *NFKB* and *APOPTOSIS* pathways from BioCarta for each sample. The expression values were normalized per gene by z-score transformation.

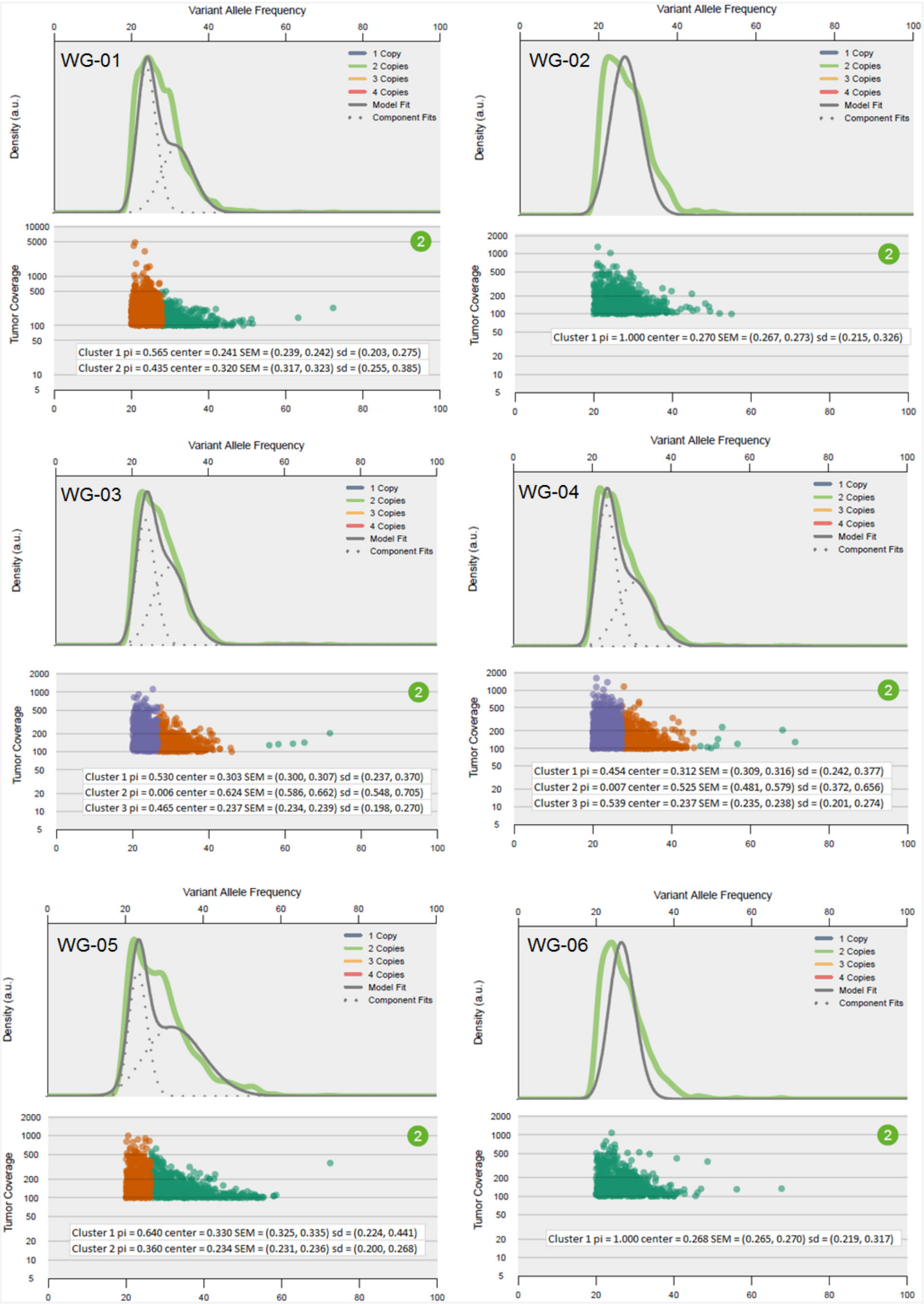
Variant Allele Frequency
Density (a.u.)
Tumor Coverage
1 Copy
2 Copies
3 Copies
4 Copies
Model Fit
Component Fits
WG-01
Cluster 1 pi = 0.565 center = 0.241 SEM = (0.239, 0.242) sd = (0.203, 0.275)
Cluster 2 pi = 0.435 center = 0.320 SEM = (0.317, 0.323) sd = (0.255, 0.385)
WG-02
Cluster 1 pi = 1.000 center = 0.270 SEM = (0.267, 0.273) sd = (0.215, 0.326)
WG-03
Cluster 1 pi = 0.530 center = 0.303 SEM = (0.300, 0.307) sd = (0.237, 0.370)
Cluster 2 pi = 0.006 center = 0.624 SEM = (0.586, 0.662) sd = (0.548, 0.705)
Cluster 3 pi = 0.465 center = 0.237 SEM = (0.234, 0.239) sd = (0.198, 0.270)
WG-04
Cluster 1 pi = 0.454 center = 0.312 SEM = (0.309, 0.316) sd = (0.242, 0.377)
Cluster 2 pi = 0.007 center = 0.525 SEM = (0.481, 0.579) sd = (0.372, 0.656)
Cluster 3 pi = 0.539 center = 0.237 SEM = (0.235, 0.238) sd = (0.201, 0.274)
WG-05
Cluster 1 pi = 0.640 center = 0.330 SEM = (0.325, 0.335) sd = (0.224, 0.441)
Cluster 2 pi = 0.360 center = 0.234 SEM = (0.231, 0.236) sd = (0.200, 0.268)
WG-06
Cluster 1 pi = 1.000 center = 0.268 SEM = (0.265, 0.270) sd = (0.219, 0.317)

(Continued)

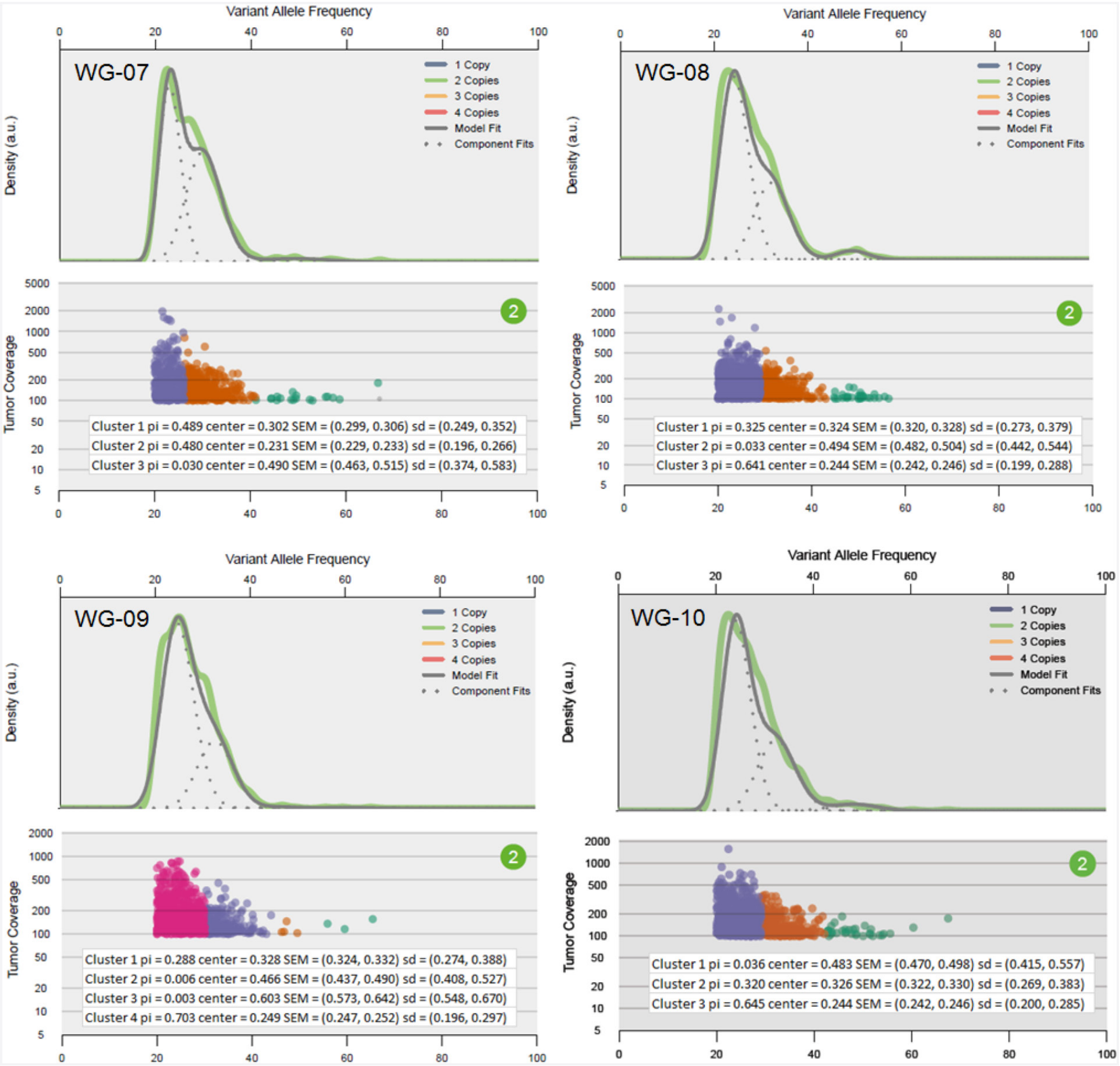

Supplementary Figure 9: Clonal architecture of tumors. We inferred clonal architecture of tumors with VAF (variant allele frequency) of heterozygous mutations in a region of copy number 2 using SciClone.

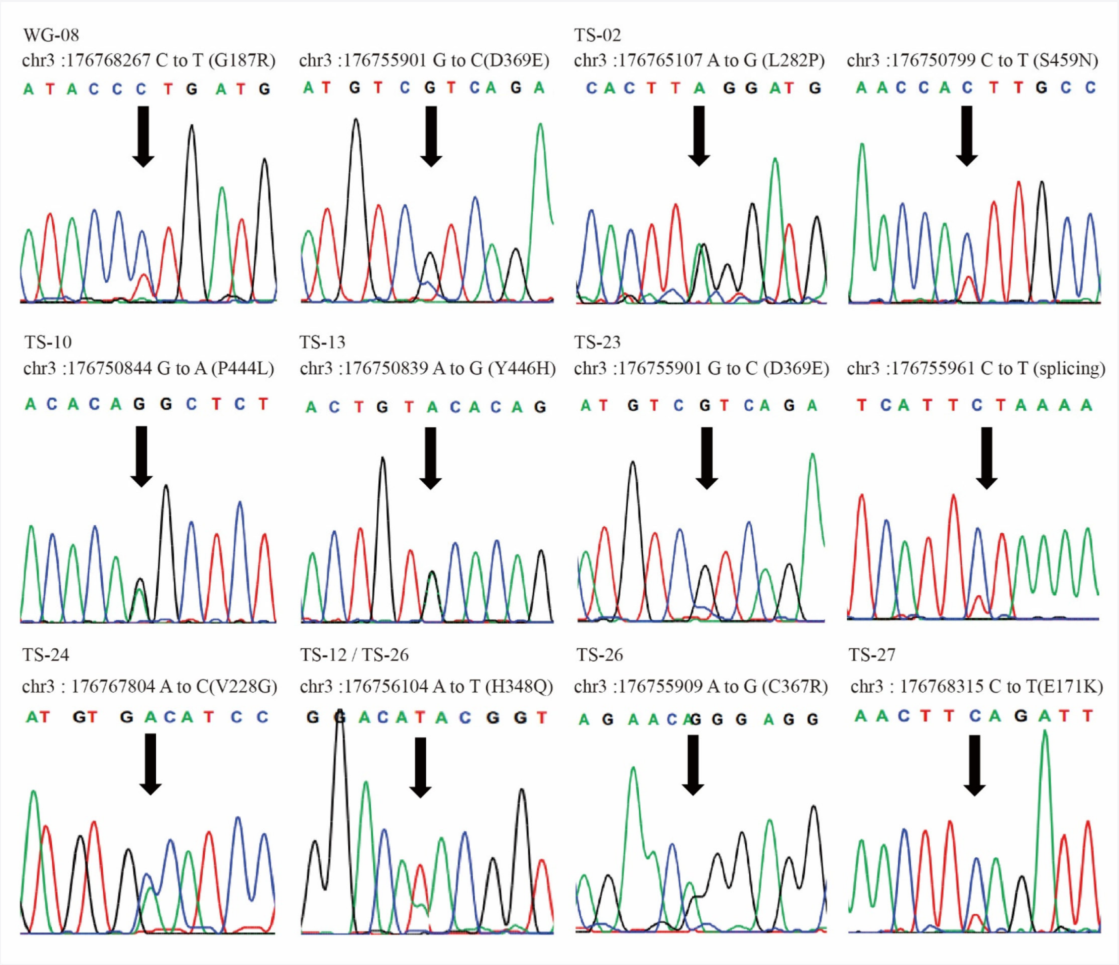

Supplementary Figure 10: Patial genomic DNA sequencing traces of TBL1XR1 mutation in Extranodal Marginal Zone Lymphoma(EMZL) patients. Patient ID and location of TBL1XR1 mutation is indicated above the chromas. The arrows show the site which the base change was occurred. The chromatograms of each patients indicate different mutation of TBL1XR1 and heterozygous type in each patients.

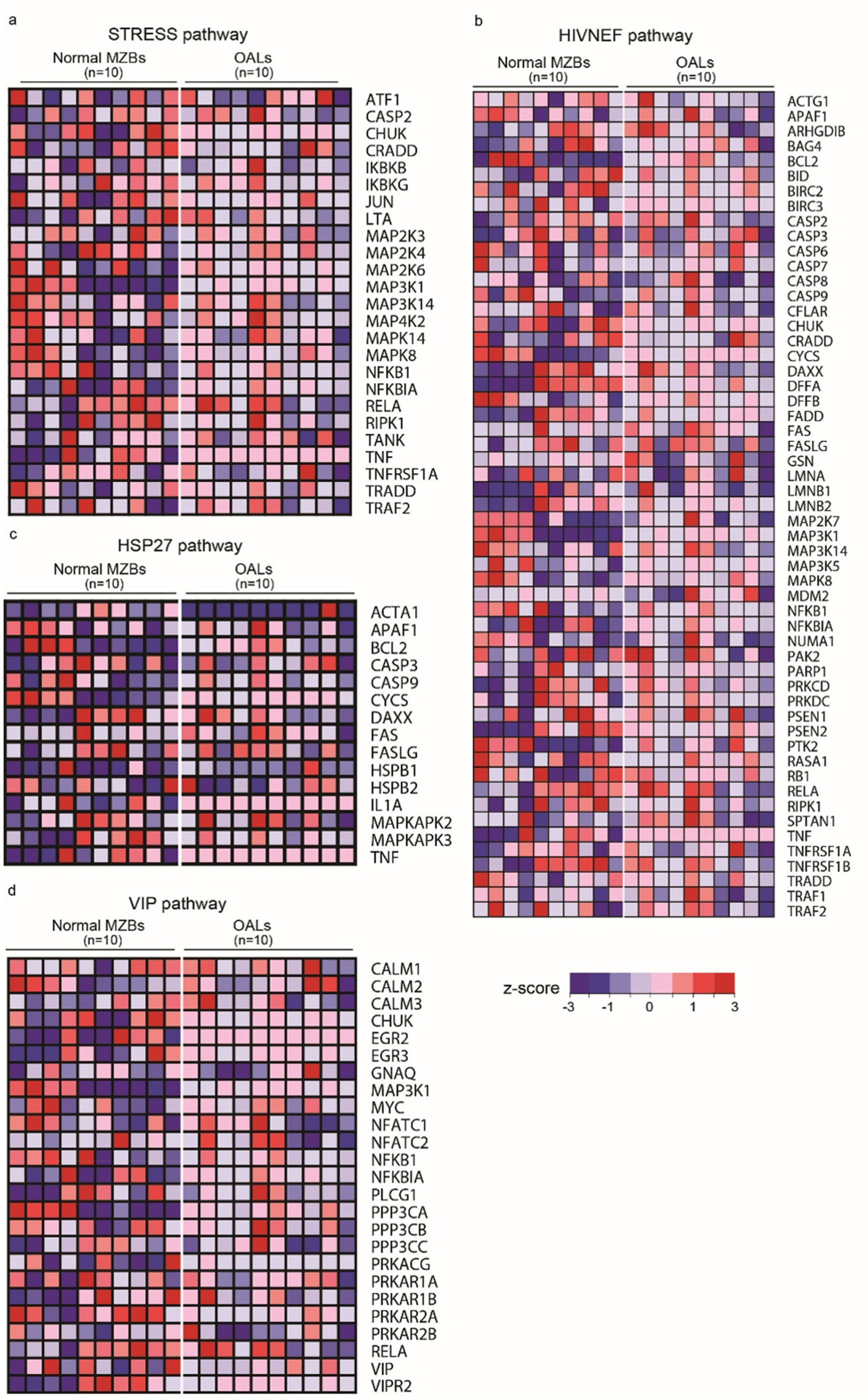
a
STRESS pathway
Normal MZBs (n=10)
OALs (n=10)
ATF1
CASP2
CHUK
CRADD
IKBKB
IKBKG
JUN
LTA
MAP2K3
MAP2K4
MAP2K6
MAP3K1
MAP3K14
MAP4K2
MAPK14
MAPK8
NFKB1
NFKBIA
RELA
RIPK1
TANK
TNF
TNFRSF1A
TRADD
TRAF2
b
HIVNEF pathway
Normal MZBs (n=10)
OALs (n=10)
ACTG1
APAF1
ARHGDIB
BAG4
BCL2
BID
BIRC2
BIRC3
CASP2
CASP3
CASP6
CASP7
CASP8
CASP9
CFLAR
CHUK
CRADD
CYCS
DAXX
DFFA
DFFB
FADD
FAS
FASLG
GSN
LMNA
LMNB1
LMNB2
MAP2K7
MAP3K1
MAP3K14
MAP3K5
MAPK8
MDM2
NFKB1
NFKBIA
NUMA1
PAK2
PARP1
PRKCD
PRKDC
PSEN1
PSEN2
PTK2
RASA1
RB1
RELA
RIPK1
SPTAN1
TNF
TNFRSF1A
TNFRSF1B
TRADD
TRAF1
TRAF2
c
HSP27 pathway
Normal MZBs (n=10)
OALs (n=10)
ACTA1
APAF1
BCL2
CASP3
CASP9
CYCS
DAXX
FAS
FASLG
HSPB1
HSPB2
IL1A
MAPKAPK2
MAPKAPK3
TNF
d
VIP pathway
Normal MZBs (n=10)
OALs (n=10)
CALM1
CALM2
CALM3
CHUK
EGR2
EGR3
GNAQ
MAP3K1
MYC
NFATC1
NFATC2
NFKB1
NFKBIA
PLCG1
PPP3CA
PPP3CB
PPP3CC
PRKACG
PRKAR1A
PRKAR1B
PRKAR2A
PRKAR2B
RELA
VIP
VIPR2
z-score
-3
-1
0
1
3

(*Continued*)

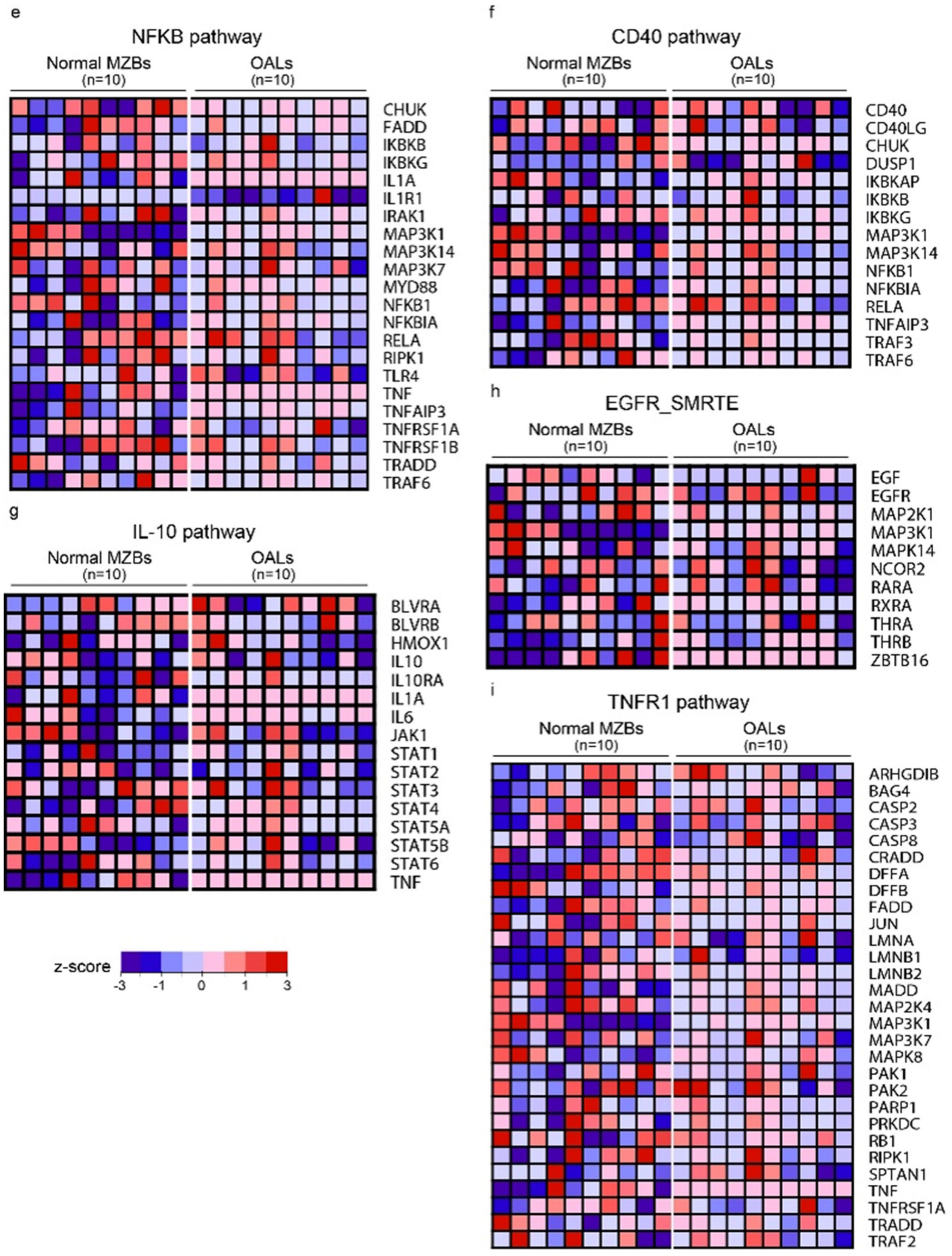

Supplementary Figure 11: Heatmap of genes in differentially expressed pathways in Figure 4. The expression values were normalized per gene by z-score transformation.

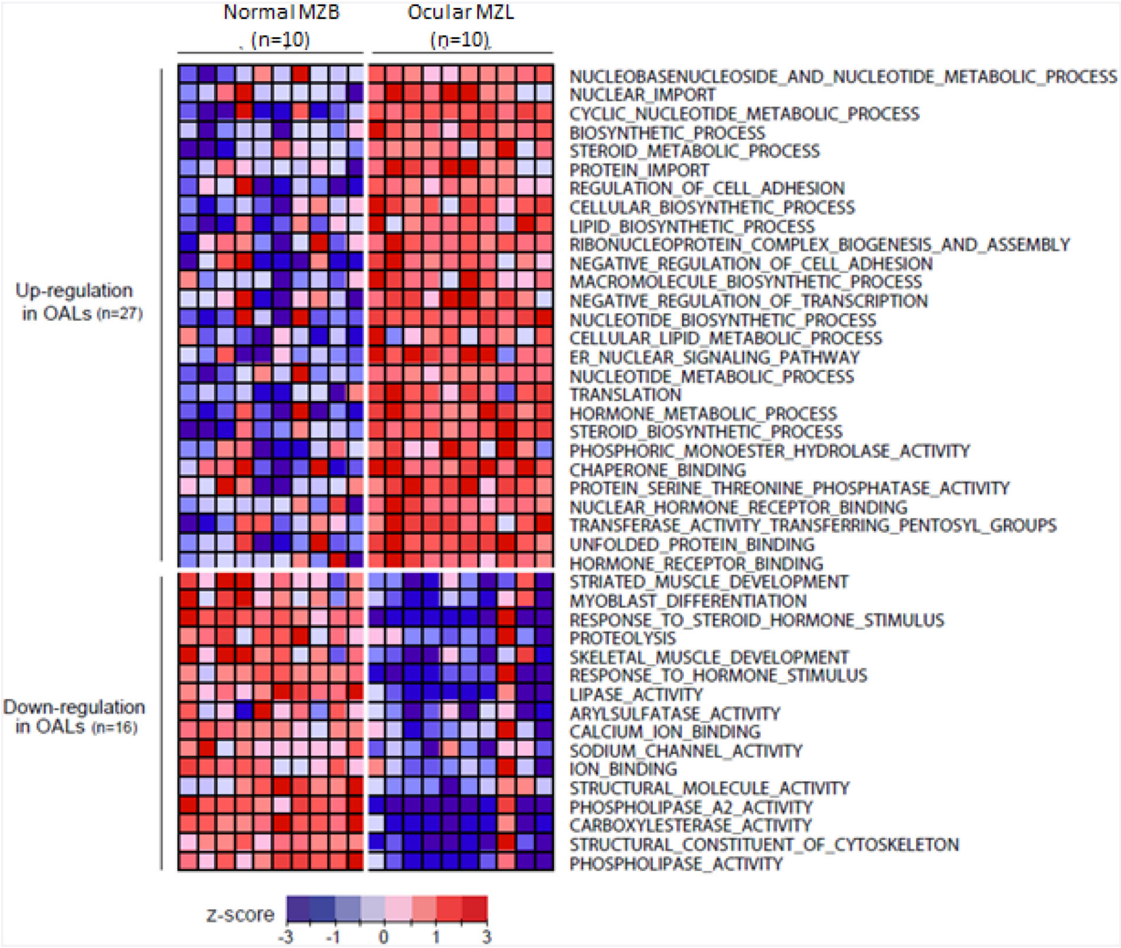

Supplementary Figure 12: Enriched gene ontology terms. ssGSEA was used to calculate enrichment score for each sample and significantly different GO terms between normal MZBs and ocular MZL were plotted (p value < 0.01, t-test). The expression values were normalized per gene by z-score transformation.
