# Supplementary material for: The mutational landscape of ocular marginal zone lymphoma identifies frequent alterations in TNFAIP3 followed by mutations in TBL1XR1 and CREBBP
Source: Oncotarget. 2017 Feb 4;8(10):17038–49. doi: 10.18632/oncotarget.14928 (PMC5370020; doi:10.18632/oncotarget.14928)
Supplement: Supplementary file 2 [file oncotarget-08-17038-s002.docx]

**Supplementary Table 1. Clinical information of patients.
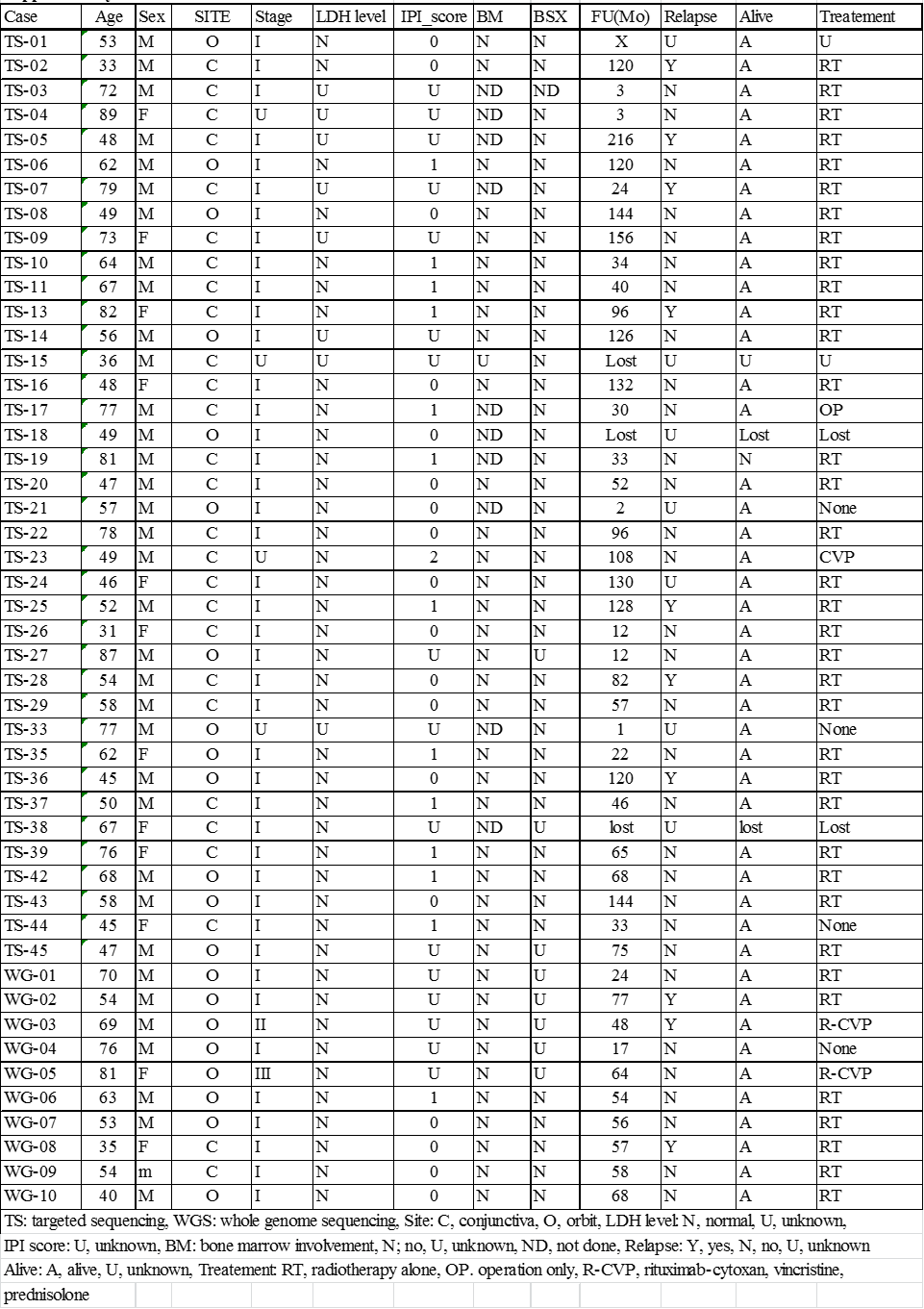
**

**Supplementary Table 2-1. Summary of WGS and Targeted-seq QC**

| **Technique** | **Sample** | **Total read** | **Depth** | **% >= 10X coverage** | **% >= 30X coverage** |
| --- | --- | --- | --- | --- | --- |
| WGS | WG-10_tumor | 1727961988 | 68.6 | 98.06% | 94.77% |
| WGS | WG-10_normal | 946828392 | 37.5 | 97.39% | 69.54% |
| WGS | WG-09_tumor | 1362702900 | 60.5 | 97.56% | 85.48% |
| WGS | WG-09_normal | 727795064 | 31.1 | 96.70% | 44.19% |
| WGS | WG-08_tumor | 1684745236 | 71.1 | 97.03% | 89.39% |
| WGS | WG-08_normal | 747025310 | 30.9 | 96.71% | 42.60% |
| WGS | WG-07_tumor | 1433528064 | 62.4 | 97.68% | 89.33% |
| WGS | WG-07_normal | 791004618 | 31.6 | 96.87% | 46.17% |
| WGS | WG-06_tumor | 1636549742 | 66.5 | 98.03% | 94.00% |
| WGS | WG-06_normal | 709535758 | 28.2 | 95.97% | 26.30% |
| WGS | WG-05_tumor | 1404132842 | 61.7 | 97.87% | 92.44% |
| WGS | WG-05_normal | 765653846 | 29.4 | 96.48% | 35.14% |
| WGS | WG-04_tumor | 1642874864 | 66.5 | 97.99% | 92.89% |
| WGS | WG-04_normal | 693441260 | 29.8 | 96.38% | 37.81% |
| WGS | WG-03_tumor | 1615132028 | 67.8 | 97.92% | 92.87% |
| WGS | WG-03_normal | 741269194 | 30 | 96.56% | 38.50% |
| WGS | WG-02_tumor | 1695046104 | 68 | 97.03% | 85.57% |
| WGS | WG-02_normal | 696991488 | 29.1 | 96.47% | 32.15% |
| WGS | WG-01_tumor | 1569201558 | 64.2 | 97.97% | 93.61% |
| WGS | WG-01_normal | 979275880 | 39.4 | 97.58% | 76.08% |

| **Technique** | **Sample** | **Total read** | **Depth** | **% >= 100X coverage** |
| --- | --- | --- | --- | --- |
| Targeted-seq | TS-01 | 14048083 | 313.77 | 87.23% |
| Targeted-seq | TS-02 | 16629691 | 347.34 | 87.91% |
| Targeted-seq | TS-03 | 15876901 | 320.96 | 87.12% |
| Targeted-seq | TS-04 | 16647963 | 270.75 | 86.24% |
| Targeted-seq | TS-05 | 17715861 | 546.11 | 88.07% |
| Targeted-seq | TS-06 | 17103223 | 232.66 | 85.18% |
| Targeted-seq | TS-07 | 16336965 | 487.44 | 88.19% |
| Targeted-seq | TS-08 | 21404747 | 604.65 | 89.65% |
| Targeted-seq | TS-09 | 22389289 | 399.78 | 88.65% |
| Targeted-seq | TS-10 | 18825901 | 311.93 | 86.70% |
| Targeted-seq | TS-11 | 19455682 | 334.4 | 87.57% |
| Targeted-seq | TS-13 | 20054619 | 532.26 | 88.05% |
| Targeted-seq | TS-14 | 21620145 | 548.97 | 89.07% |
| Targeted-seq | TS-15 | 20433356 | 367.81 | 86.70% |
| Targeted-seq | TS-16 | 18299792 | 297.46 | 87.40% |
| Targeted-seq | TS-17 | 23311239 | 517.59 | 88.97% |
| Targeted-seq | TS-18 | 19508760 | 222.6 | 82.91% |
| Targeted-seq | TS-19 | 18432518 | 235.62 | 83.98% |
| Targeted-seq | TS-20 | 17242273 | 565.21 | 87.19% |
| Targeted-seq | TS-21 | 16008406 | 220.66 | 84.78% |
| Targeted-seq | TS-22 | 17654325 | 436.68 | 88.77% |
| Targeted-seq | TS-23 | 15882652 | 416.26 | 88.25% |
| Targeted-seq | TS-24 | 17891124 | 595.88 | 88.71% |
| Targeted-seq | TS-25 | 15746994 | 392.13 | 86.56% |
| Targeted-seq | TS-26 | 13426158 | 294.12 | 86.75% |
| Targeted-seq | TS-27 | 19473678 | 390.23 | 86.05% |
| Targeted-seq | TS-28 | 19074354 | 263.69 | 86.81% |
| Targeted-seq | TS-29 | 19513257 | 503.04 | 86.80% |
| Targeted-seq | TS-33 | 21142561 | 441.28 | 87.24% |
| Targeted-seq | TS-35 | 14963021 | 456.22 | 88.64% |
| Targeted-seq | TS-36 | 17879534 | 723.36 | 89.16% |
| Targeted-seq | TS-37 | 21446143 | 385.36 | 87.28% |
| Targeted-seq | TS-38 | 21534141 | 498.69 | 86.96% |
| Targeted-seq | TS-39 | 20903894 | 657.51 | 89.59% |
| Targeted-seq | TS-42 | 21667129 | 351.85 | 87.73% |
| Targeted-seq | TS-43 | 22516179 | 666.26 | 90.00% |
| Targeted-seq | TS-44 | 16769091 | 343.34 | 84.78% |
| Targeted-seq | TS-45 | 16921895 | 219.53 | 84.13% |

**Supplementary Table 2-2. Summary of RNA-seq QC**

| **Technique** | **Sample** | **Number of reads** | **Number of aligned reads** | **The percentage of bases mapping to mRNA divided by the number of aligned bases** |
| --- | --- | --- | --- | --- |
| RNA-seq | WG-01_tumor | 71,628,964 | 59,798,689 | 90.07% |
| RNA-seq | WG-02_tumor | 67,764,220 | 57,877,372 | 91.36% |
| RNA-seq | WG-03_tumor | 69,250,988 | 48,785,169 | 88.79% |
| RNA-seq | WG-04_tumor | 77,401,886 | 59,329,861 | 88.70% |
| RNA-seq | WG-05_tumor | 69,227,296 | 46,471,777 | 82.24% |
| RNA-seq | WG-06_tumor | 68,248,502 | 49,661,017 | 87.95% |
| RNA-seq | WG-07_tumor | 79,796,544 | 63,065,368 | 77.70% |
| RNA-seq | WG-08_tumor | 77,969,612 | 65,816,188 | 88.36% |
| RNA-seq | WG-09_tumor | 66,217,202 | 54,672,947 | 89.71% |
| RNA-seq | WG-10_tumor | 79,477,588 | 59,597,122 | 86.25% |

**Supplementary Table 3. List of identified CNVs**

| sample | chrom | start | end | len | binNum | tumor | tumor_expect | normal | normal_expect | log2.copyRatio | log2.TumorExpectRatio | Type |
| --- | --- | --- | --- | --- | --- | --- | --- | --- | --- | --- | --- | --- |
| WG-02 | chr11 | 50314522 | 55025617 | 4711095 | 9721 | 238005 | 477312 | 283419 | 266838 | -1.128258176 | -1.000204636 | deletion |
| WG-01 | chr3 | 60124 | 197962382 | 197902258 | 1874989 | 132551903 | 107208204.7 | 58238802 | 58100132 | 0.29705629 | 0.340674395 | amplification |
| WG-03 | chr3 | 60124 | 197962382 | 197902258 | 1874991 | 111847000 | 86388600 | 48631300 | 48396800 | 0.350075468 | 0.398283767 | amplification |
| WG-05 | chr3 | 60124 | 197962382 | 197902258 | 1874992 | 130822700 | 97833800 | 43459400 | 43189200 | 0.385842069 | 0.401781658 | amplification |
| WG-04 | chr3 | 60124 | 197962382 | 197902258 | 1874520 | 129774615 | 95928603.4 | 60573300 | 60455199.2 | 0.407788811 | 0.450981979 | amplification |
| WG-02 | chr12 | 34561238 | 38419409 | 3858171 | 7649 | 159995 | 369679 | 222133 | 209033 | -1.333284395 | -1.204510182 | deletion |
| WG-03 | chr15 | 22699486 | 102521344 | 79821858 | 736572 | 42777600 | 33292200 | 18273600 | 18410200 | 0.356851009 | 0.38734139 | amplification |
| WG-05 | chr18 | 10001 | 78017200 | 78007199 | 717327 | 51429700 | 37730000 | 16645900 | 16486500 | 0.408639639 | 0.429463286 | amplification |
| WG-08 | chrY | 2649424 | 58912807 | 56263383 | 115959 | 17731 | 6304940 | 6115 | 2879990 | 0.397508374 | -8.475619435 | amplification |
| WG-07 | chr13 | 53889710 | 109995545 | 56105835 | 544844 | 34624800 | 30334140 | 13301460 | 13026720 | 0.125151654 | 0.183280509 | amplification |
| WG-01 | chr6 | 63764 | 45833716 | 45769952 | 441008 | 29679637 | 24374864.7 | 13300480 | 13396412.6 | 0.288801105 | 0.318611569 | amplification |
| WG-07 | chr21 | 11186814 | 32557632 | 21370818 | 170363 | 10961100 | 9601580 | 4141020 | 4070620 | 0.130710293 | 0.183466034 | amplification |
| WG-06 | chr15 | 61278264 | 78026805 | 16748541 | 161973 | 11646800 | 7855390 | 3707340 | 3808500 | 0.598797557 | 0.600359557 | amplification |
| WG-04 | chr15 | 73798351 | 76267444 | 2469093 | 22883 | 1432100 | 1058710 | 700671 | 707044 | 0.42351674 | 0.450831503 | amplification |
| WG-05 | chr14 | 106330081 | 106993912 | 663831 | 5601 | 86533 | 323625 | 119848 | 122653 | -1.893992325 | -1.920427087 | deletion |
| WG-04 | chr6 | 137341111 | 138360509 | 1019398 | 10015 | 147155 | 513080 | 325853 | 321648 | -1.845956631 | -1.786840497 | deletion |
| WG-05 | chr6 | 137354725 | 138897819 | 1543094 | 15145 | 256939 | 856188 | 344135 | 345190 | -1.756453961 | -1.753928078 | deletion |
| WG-02 | chr12 | 109121313 | 133841505 | 24720192 | 238878 | 10606900 | 11484400 | 5780430 | 5838620 | -0.137566919 | -0.110936324 | deletion |
| WG-07 | chr19 | 27738975 | 28124644 | 385669 | 3598 | 138125 | 218641 | 153434 | 85896.9 | -1.535130677 | -0.670172317 | deletion |
| WG-02 | chr15 | 22699486 | 102521344 | 79821858 | 736572 | 41713700 | 35599400 | 18956000 | 18933600 | 0.189617435 | 0.232404448 | amplification |
| WG-02 | chr16 | 60001 | 90294705 | 90234704 | 712078 | 32145594 | 33836093 | 17698119 | 17671102 | -0.113491113 | -0.070205893 | deletion |
| WG-10 | chr19 | 27739075 | 28118444 | 379369 | 3535 | 74712 | 144728 | 121638 | 89975.7 | -1.402502664 | -0.927050269 | deletion |
| WG-02 | chr17 | 1 | 81195112 | 81195111 | 720982 | 31936900 | 33890600 | 17526800 | 17706300 | -0.108305848 | -0.081924807 | deletion |
| WG-02 | chr19 | 27738975 | 28125144 | 386169 | 3603 | 76003 | 174805 | 134508 | 99795.9 | -1.669602817 | -1.19788209 | deletion |
| WG-07 | chr5 | 45906924 | 49554528 | 3647604 | 5738 | 160052 | 346940 | 153994 | 136672 | -1.32390285 | -1.123728297 | deletion |
| WG-07 | chr7 | 61971130 | 62452629 | 481499 | 4506 | 124745 | 274306 | 117717 | 107662 | -1.301218676 | -1.144387006 | deletion |
| WG-05 | chr19 | 27738975 | 28124444 | 385469 | 3596 | 92155 | 169595 | 110814 | 87638.4 | -1.242832676 | -0.897385641 | deletion |
| WG-07 | chr12 | 34560638 | 38422009 | 3861371 | 7681 | 214052 | 456035 | 195909 | 180903 | -1.241751519 | -1.098766056 | deletion |
| WG-10 | chr5 | 45907738 | 49552783 | 3645045 | 5718 | 111980 | 235134 | 158610 | 145265 | -1.210622485 | -1.043360175 | deletion |
| WG-10 | chr7 | 61971130 | 62453535 | 482405 | 4515 | 90099 | 191519 | 123230 | 114325 | -1.209700941 | -1.061022621 | deletion |
| WG-02 | chr19 | 70830 | 24629274 | 24558444 | 230757 | 8524000 | 10280200 | 5145880 | 5348210 | -0.25197264 | -0.266529744 | deletion |
| WG-05 | chr7 | 61971130 | 62453329 | 482199 | 4513 | 107115 | 223263 | 112021 | 108944 | -1.124134405 | -1.07701001 | deletion |
| WG-05 | chr5 | 45906224 | 46405517 | 499293 | 4694 | 108059 | 221609 | 116497 | 114154 | -1.089876906 | -1.053623589 | deletion |
| WG-10 | chr12 | 34560438 | 38441336 | 3880898 | 7853 | 171203 | 331128 | 212507 | 197348 | -1.072033309 | -0.924799122 | deletion |
| WG-07 | chr11 | 50327752 | 55027170 | 4699418 | 9694 | 316941 | 582954 | 257051 | 230679 | -1.070936635 | -0.886750564 | deletion |
| WG-05 | chrX | 58319370 | 62041137 | 3721767 | 5177 | 125487 | 256061 | 120107 | 125038 | -0.995271529 | -1.046375975 | deletion |
| WG-10 | chr11 | 50303957 | 55026417 | 4722460 | 9730 | 229840 | 410181 | 268765 | 245940 | -0.977253463 | -0.808748863 | deletion |
| WG-05 | chr12 | 34560338 | 38419309 | 3858971 | 7657 | 200130 | 374903 | 186703 | 183285 | -0.95660459 | -0.923006275 | deletion |
| WG-06 | chr6 | 136898841 | 140386142 | 3487301 | 34149 | 965238 | 1814810 | 842167 | 850906 | -0.904188199 | -0.878681154 | deletion |
| WG-02 | chr19 | 28125145 | 59118885 | 30993740 | 292872 | 11947470 | 13561845.9 | 6799212 | 6995789.97 | -0.179074095 | -0.179112322 | deletion |
| WG-05 | chr11 | 50303957 | 55027470 | 4723513 | 9739 | 278421 | 478444 | 242144 | 234489 | -0.851795329 | -0.798508394 | deletion |
| WG-07 | chr8 | 43427603 | 47456113 | 4028510 | 9254 | 321514 | 503363 | 224051 | 202868 | -0.825604403 | -0.654300433 | deletion |
| WG-03 | chr14 | 106329781 | 107289492 | 959711 | 8372 | 227868 | 375717 | 218267 | 208063 | -0.806086393 | -0.695778001 | deletion |
| WG-07 | chr6 | 136918807 | 139213197 | 2294390 | 22487 | 788559 | 1257590 | 517018 | 515431 | -0.713407182 | -0.680953862 | deletion |
| WG-04 | chr6 | 138360510 | 139809221 | 1448711 | 14115 | 456648 | 719498 | 449902 | 451912 | -0.674847921 | -0.640901432 | deletion |
| WG-05 | chr7 | 127906373 | 131017337 | 3110964 | 28474 | 1110970 | 1726200 | 598269 | 617128 | -0.615372603 | -0.65320612 | deletion |
| WG-05 | chr6 | 138897820 | 143735521 | 4837701 | 46967 | 1506200 | 2239700 | 1114290 | 1111620 | -0.600221484 | -0.589818503 | deletion |
| WG-03 | chr6 | 131959837 | 159951429 | 27991592 | 271969 | 8537960 | 12599300 | 7051390 | 7033760 | -0.580556584 | -0.535710217 | deletion |
| WG-06 | chr6 | 70343197 | 74322310 | 3979113 | 38160 | 1631830 | 2111350 | 988332 | 982247 | -0.388804606 | -0.339494245 | deletion |
| WG-05 | chrY | 2649424 | 58967660 | 56318236 | 117138 | 16740 | 6146900.2 | 9200 | 2711090.2 | -0.341760508 | -8.537842023 | deletion |
| WG-05 | chr14 | 106993913 | 107289492 | 295579 | 2768 | 132801 | 158393 | 63096 | 62194.7 | -0.29936775 | -0.271668921 | deletion |
| WG-02 | chr22 | 16051156 | 51244518 | 35193362 | 317945 | 13414209 | 14630917 | 7481136 | 7537579.8 | -0.151759302 | -0.121522119 | deletion |
| WG-07 | chr7 | 62452630 | 76170618 | 13717988 | 108210 | 4591160 | 5499440 | 2241240 | 2331250 | -0.239220368 | -0.268008827 | deletion |
| WG-07 | chr7 | 10001 | 7071542 | 7061541 | 64598 | 2501110 | 2951260 | 1232500 | 1275010 | -0.225442538 | -0.246345334 | deletion |
| WG-07 | chr4 | 68575 | 9237793 | 9169218 | 85249 | 3493835 | 3923715.6 | 1708877 | 1694298.68 | -0.215370274 | -0.174991817 | deletion |
| WG-07 | chr19 | 70830 | 24629274 | 24558444 | 230757 | 8871190 | 10595600 | 4317400 | 4598700 | -0.200803353 | -0.263848555 | deletion |
| WG-07 | chr13 | 109995546 | 115109830 | 5114284 | 45846 | 2040370 | 2226180 | 977935 | 952514 | -0.199338673 | -0.133322272 | deletion |
| WG-02 | chr3 | 60124 | 197962382 | 197902258 | 1874998 | 108678854 | 88549717.8 | 49867400 | 49668496.9 | 0.252400639 | 0.299247733 | amplification |
| WG-02 | chr5 | 45907324 | 49552783 | 3645459 | 5722 | 114945 | 277205 | 171060 | 158166 | -1.420417614 | -1.266273479 | deletion |
| WG-07 | chr12 | 109128906 | 133841505 | 24712599 | 238804 | 10603100 | 11866800 | 4978340 | 5051450 | -0.177012962 | -0.170027645 | deletion |
| WG-07 | chr19 | 28580573 | 59118885 | 30538312 | 288558 | 11944800 | 13769300 | 5640520 | 5942460 | -0.165441342 | -0.212655338 | deletion |
| WG-07 | chr2 | 230774583 | 243189325 | 12414742 | 117995 | 5322220 | 5744470 | 2468460 | 2456880 | -0.152530456 | -0.117728463 | deletion |
| WG-02 | chr7 | 61971130 | 62453535 | 482405 | 4515 | 92022 | 224643 | 134233 | 124065 | -1.438571711 | -1.2838473 | deletion |
| WG-07 | chr22 | 16051156 | 51244518 | 35193362 | 317945 | 13742348 | 15084533.1 | 6355456 | 6496489.65 | -0.138377726 | -0.142024339 | deletion |
| WG-02 | chr7 | 62453536 | 76672148 | 14218612 | 109360 | 4616510 | 5287120 | 2628560 | 2720680 | -0.183329952 | -0.19194337 | deletion |
| WG-10 | chrY | 2649424 | 28815077 | 26165653 | 117745 | 2397840 | 5184450 | 1454490 | 2889760 | -0.135603065 | -1.085573646 | deletion |
| WG-07 | chr21 | 32557633 | 48119847 | 15562214 | 151260 | 7086770 | 7575690 | 3198780 | 3207860 | -0.1277606 | -0.103831887 | deletion |
| WG-02 | chr8 | 43427303 | 47456113 | 4028810 | 9257 | 258764 | 428947 | 255253 | 236665 | -0.875589142 | -0.725426393 | deletion |
| WG-07 | chr16 | 60001 | 90294705 | 90234704 | 712145 | 34007932 | 36165231 | 15253565 | 15304855 | -0.119490112 | -0.096314906 | deletion |
| WG-06 | chr19 | 70830 | 59118885 | 59048055 | 525048 | 20271800 | 22868100 | 11082400 | 11578300 | -0.118928933 | -0.141681567 | deletion |
| WG-07 | chr14 | 88058577 | 107289492 | 19230915 | 186309 | 8780550 | 9270180 | 3953610 | 3941210 | -0.118418922 | -0.08586886 | deletion |
| WG-05 | chr19 | 70830 | 24629274 | 24558444 | 230757 | 13120200 | 14661700 | 4353420 | 4562600 | -0.116923991 | -0.177689032 | deletion |
| WG-02 | chrX | 58319070 | 62040937 | 3721867 | 5178 | 55104 | 257804 | 74895 | 142394 | -1.336443757 | -2.222309605 | deletion |
| WG-07 | chr1 | 10027 | 47942671 | 47932644 | 441401 | 19409100 | 21108100 | 8792700 | 9086640 | -0.109223764 | -0.12864632 | deletion |
| WG-02 | chrY | 2649424 | 28814954 | 26165530 | 117853 | 2657810 | 5656160 | 1565260 | 3133520 | -0.125548181 | -1.085848868 | deletion |
| WG-08 | chr19 | 70830 | 59118885 | 59048055 | 527232 | 27405300 | 30637700 | 10992600 | 11465500 | -0.108008675 | -0.162407629 | deletion |
| WG-07 | chrY | 2649424 | 28814954 | 26165530 | 117746 | 3169410 | 6563720 | 1383410 | 2728530 | -0.106005218 | -1.057882214 | deletion |

**Supplementary Table 5. List of identified SNVs**

| Sample | Chr | Start | End | Ref | Alt | Gene | Function | Transcript | AAChange | in-silico prediction | | |
| --- | --- | --- | --- | --- | --- | --- | --- | --- | --- | --- | --- | --- |
|  |  |  |  |  |  |  |  |  |  | SIFT | PolyPhen | MutationTaster |
| TS-17 | chr16 | 89347240 | 89347240 | C | T | ANKRD11 | missense SNV | NM_001256183 | p.E1904K | T | P | D |
| TS-29 | chr16 | 89349466 | 89349466 | C | T | ANKRD11 | missense SNV | NM_001256183 | p.D1162N | D | B | D |
| TS-05 | chr5 | 112178256 | 112178256 | A | G | APC | missense SNV | NM_001127511 | p.Q2304R | . | D | D |
| TS-25 | chr5 | 112177902 | 112177902 | G | T | APC | missense SNV | NM_001127511 | p.R2186L | . | D | D |
| TS-33 | chr3 | 52437500 | 52437500 | C | G | BAP1 | missense SNV | NM_004656 | p.G554A | D | D | D |
| WG-05 | chr3 | 52443754 | 52443754 | A | G | BAP1 | missense SNV | NM_004656 | p.F15L | D | D | D |
| TS-19 | chr1 | 85733386 | 85733386 | A | C | BCL10 | stopgain SNV | NM_003921 | p.L209X | . | . | . |
| TS-27 | chr1 | 85733576 | 85733576 | T | A | BCL10 | stopgain SNV | NM_003921 | p.K146X | . | . | . |
| TS-16 | chr19 | 15366982 | 15366982 | T | - | BRD4 | frameshift deletion | NM_014299 | p.K548fs | . | . | . |
| TS-20 | chr19 | 15379723 | 15379723 | T | G | BRD4 | missense SNV | NM_014299 | p.Y139S | D | D | D |
| TS-27 | chr19 | 15379714 | 15379714 | A | C | BRD4 | splice site | NM_058243 | . | . | . | . |
| WG-09 | chr7 | 2977602 | 2977602 | T | C | CARD11 | missense SNV | NM_032415 | p.Y361C | D | D | D |
| TS-22 | chr7 | 2979459 | 2979469 | TTCTTGGGCCG | - | CARD11 | frameshift deletion | NM_032415 | p.260_263del | . | . | . |
| TS-22 | chr7 | 2979471 | 2979471 | T | - | CARD11 | frameshift deletion | NM_032415 | p.N259fs | . | . | . |
| TS-09 | chr17 | 62006799 | 62006799 | A | T | CD79B | missense SNV | NM_021602 | p.Y92N | D | D | D |
| TS-06 | chr17 | 62007514 | 62007514 | T | C | CD79B | missense SNV | NM_000626 | p.N117S | T | D | D |
| WG-03 | chr16 | 3841988 | 3841988 | G | A | CREBBP | stopgain SNV | NM_004380 | p.Q442X | . | . | . |
| TS-02 | chr16 | 3788614 | 3788614 | G | A | CREBBP | missense SNV | NM_001079846 | p.T1409I | D | D | D |
| TS-09 | chr16 | 3788617 | 3788617 | C | T | CREBBP | missense SNV | NM_001079846 | p.R1408H | D | D | D |
| TS-28 | chr16 | 3823754 | 3823760 | GTGACAC | - | CREBBP | frameshift deletion | NM_001079846 | p.781_783del | . | . | . |
| TS-21 | chr16 | 3807902 | 3807902 | G | A | CREBBP | stopgain SNV | NM_001079846 | p.R1135X | . | . | . |
| TS-11 | chr16 | 3900786 | 3900786 | G | A | CREBBP | stopgain SNV | NM_001079846 | p.Q104X | . | . | . |
| TS-29 | chr16 | 3779500 | 3779500 | G | A | CREBBP | missense SNV | NM_001079846 | p.L1812F | T | D | D |
| TS-29 | chr16 | 3779501 | 3779501 | C | A | CREBBP | missense SNV | NM_001079846 | p.K1811N | D | D | D |
| TS-02 | chr7 | 101891841 | 101891841 | G | A | CUX1 | missense SNV | NM_001202543 | p.G1357D | T | P | D |
| TS-17 | chr7 | 101845359 | 101845359 | C | T | CUX1 | missense SNV | NM_001202543 | p.P939S | D | D | D |
| TS-02 | chr2 | 136872544 | 136872544 | G | - | CXCR4 | frameshift deletion | NM_001008540 | p.T322fs | . | . | . |
| TS-21 | chr2 | 136872475 | 136872475 | G | - | CXCR4 | frameshift deletion | NM_001008540 | p.S345fs | . | . | . |
| TS-24 | chr15 | 99434707 | 99434707 | C | G | IGF1R | missense SNV | NM_000875 | p.P265R | D | D | D |
| TS-26 | chr15 | 99465574 | 99465574 | G | A | IGF1R | missense SNV | NM_000875 | p.R800H | D | D | D |
| TS-02 | chr2 | 141458142 | 141458142 | A | C | LRP1B | missense SNV | NM_018557 | p.L2159R | D | P | D |
| TS-22 | chr2 | 142238063 | 142238063 | A | C | LRP1B | missense SNV | NM_018557 | p.I82S | D | B | D |
| TS-27 | chr2 | 141032054 | 141032054 | A | T | LRP1B | missense SNV | NM_018557 | p.C4361S | T | D | D |
| TS-25 | chr7 | 116371899 | 116371899 | G | A | MET | missense SNV | NM_000245 | p.G460S | D | D | D |
| TS-36 | chr7 | 116411986 | 116411986 | C | T | MET | missense SNV | NM_000245 | p.P991S | T | D | D |
| TS-13 | chr12 | 49425794 | 49425794 | G | A | KMT2D | stopgain SNV | NM_003482 | p.Q4232X | . | . | . |
| TS-13 | chr12 | 49425799 | 49425821 | TGCCGCTGCATGAGGAGTGCCTG | - | KMT2D | frameshift deletion | NM_003482 | p.4223_4230del | . | . | . |
| TS-26 | chr12 | 49440462 | 49440462 | T | C | KMT2D | missense SNV | NM_003482 | p.I1450V | D | B | D |
| TS-02 | chr12 | 49423021 | 49423021 | T | A | KMT2D | splice site | NM_003482 | . | . | . | . |
| TS-09 | chr11 | 94209474 | 94209474 | A | G | MRE11A | missense SNV | NM_005590 | p.F214L | T | P | D |
| TS-20 | chr11 | 94224067 | 94224067 | C | A | MRE11A | missense SNV | NM_005590 | p.D29Y | D | D | D |
| TS-02 | chr3 | 38182292 | 38182292 | G | A | MYD88 | missense SNV | NM_001172566 | p.A138T | T | D | D |
| TS-07 | chr3 | 38182736 | 38182736 | T | G | MYD88 | missense SNV | NM_001172568 | p.W252G | T | D | D |
| WG-01 | chr17 | 29557392 | 29557392 | G | T | NF1 | missense SNV | NM_000267 | p.M1035I | T | P | D |
| TS-38 | chr17 | 29701039 | 29701039 | A | C | NF1 | missense SNV | NM_000267 | p.K2775Q | D | D | D |
| TS-05 | chr9 | 139391998 | 139391998 | G | C | NOTCH1 | missense SNV | NM_017617 | p.L2065V | D | B | D |
| TS-20 | chr9 | 139400036 | 139400036 | G | A | NOTCH1 | missense SNV | NM_017617 | p.R1438C | T | P | D |
| TS-35 | chr9 | 139413893 | 139413893 | G | A | NOTCH1 | splice site | NM_017617 | . | . | . | . |
| TS-10 | chr19 | 42603714 | 42603721 | GAAGAGCT | - | POU2F2 | frameshift deletion | NM_001207025 | p.153_156del | . | . | . |
| TS-39 | chr19 | 42596244 | 42596244 | C | T | POU2F2 | missense SNV | NM_001247994 | p.C398Y | D | D | D |
| TS-23 | chr12 | 15654584 | 15654584 | G | A | PTPRO | missense SNV | NM_002848 | p.R231H | T | B | D |
| TS-21 | chr12 | 15710447 | 15710447 | C | T | PTPRO | missense SNV | NM_030668 | p.P62S | T | D | D |
| WG-08 | chr3 | 176755901 | 176755901 | G | C | TBL1XR1 | missense SNV | NM_024665 | p.D369E | T | D | D |
| WG-08 | chr3 | 176768267 | 176768267 | C | T | TBL1XR1 | missense SNV | NM_024665 | p.G187R | D | D | D |
| TS-23 | chr3 | 176755901 | 176755901 | G | C | TBL1XR1 | missense SNV | NM_024665 | p.D369E | T | D | D |
| TS-02 | chr3 | 176750799 | 176750799 | C | T | TBL1XR1 | missense SNV | NM_024665 | p.S459N | D | D | D |
| TS-02 | chr3 | 176765107 | 176765107 | A | G | TBL1XR1 | missense SNV | NM_024665 | p.L282P | T | D | D |
| TS-10 | chr3 | 176750844 | 176750844 | G | A | TBL1XR1 | missense SNV | NM_024665 | p.P444L | T | P | D |
| TS-24 | chr3 | 176767804 | 176767804 | A | C | TBL1XR1 | missense SNV | NM_024665 | p.V228G | D | D | D |
| TS-13 | chr3 | 176750839 | 176750839 | A | G | TBL1XR1 | missense SNV | NM_024665 | p.Y446H | T | D | D |
| TS-25 | chr3 | 176767804 | 176767804 | A | C | TBL1XR1 | missense SNV | NM_024665 | p.V228G | D | D | D |
| TS-26 | chr3 | 176755909 | 176755909 | A | G | TBL1XR1 | missense SNV | NM_024665 | p.C367R | D | D | D |
| TS-26 | chr3 | 176756104 | 176756104 | A | T | TBL1XR1 | missense SNV | NM_024665 | p.H348Q | D | D | D |
| TS-27 | chr3 | 176768315 | 176768315 | C | T | TBL1XR1 | missense SNV | NM_024665 | p.E171K | T | D | D |
| TS-23 | chr3 | 176755961 | 176755961 | C | T | TBL1XR1 | splice site | NM_024665 | . | . | . | . |
| WG-01 | chr6 | 138200107 | 138200107 | - | C | TNFAIP3 | frameshift insertion | NM_001270507 | p.A509fs | . | . | . |
| WG-03 | chr6 | 138199933 | 138199933 | G | T | TNFAIP3 | stopgain SNV | NM_001270507 | p.E451X | . | . | . |
| WG-02 | chr6 | 138196087 | 138196088 | AC | - | TNFAIP3 | frameshift deletion | NM_001270507 | p.134_134del | . | . | . |
| TS-01 | chr6 | 138196895 | 138196895 | T | - | TNFAIP3 | frameshift deletion | NM_001270507 | p.L186fs | . | . | . |
| TS-02 | chr6 | 138196134 | 138196134 | C | T | TNFAIP3 | stopgain SNV | NM_001270507 | p.Q150X | . | . | . |
| TS-02 | chr6 | 138200156 | 138200177 | TCCAGGATGTTACCAGGACATT | - | TNFAIP3 | frameshift deletion | NM_001270507 | p.525_532del | . | . | . |
| TS-09 | chr6 | 138197274 | 138197274 | C | G | TNFAIP3 | missense SNV | NM_001270507 | p.P259R | D | D | D |
| TS-03 | chr6 | 138199666 | 138199666 | C | T | TNFAIP3 | stopgain SNV | NM_001270507 | p.Q362X | . | . | . |
| TS-04 | chr6 | 138199825 | 138199825 | C | T | TNFAIP3 | stopgain SNV | NM_001270507 | p.Q415X | . | . | . |
| TS-04 | chr6 | 138199844 | 138199886 | TGAACTCCAAGCCGGGCCCTGAGGGGCTCCCTGGCATGGCGCT | - | TNFAIP3 | frameshift deletion | NM_001270507 | p.421_435del | . | . | . |
| TS-10 | chr6 | 138197143 | 138197143 | A | - | TNFAIP3 | frameshift deletion | NM_001270507 | p.L215fs | . | . | . |
| TS-11 | chr6 | 138192659 | 138192663 | GGTAA | - | TNFAIP3 | frameshift deletion | NM_001270507 | p.99_99del | . | . | . |
| TS-11 | chr6 | 138199666 | 138199666 | C | - | TNFAIP3 | frameshift deletion | NM_001270507 | p.Q362fs | . | . | . |
| TS-05 | chr6 | 138199675 | 138199675 | - | G | TNFAIP3 | frameshift insertion | NM_001270507 | p.R365fs | . | . | . |
| TS-05 | chr6 | 138199946 | 138199964 | GGGGGCCTCATTCGGCCCC | - | TNFAIP3 | frameshift deletion | NM_001270507 | p.455_461del | . | . | . |
| TS-06 | chr6 | 138198363 | 138198370 | ATGGCACA | - | TNFAIP3 | frameshift deletion | NM_001270507 | p.319_321del | . | . | . |
| TS-13 | chr6 | 138202170 | 138202171 | AG | - | TNFAIP3 | splice site | NM_001270507 | . | . | . | . |
| TS-14 | chr6 | 138196014 | 138196014 | C | T | TNFAIP3 | stopgain SNV | NM_001270507 | p.Q110X | . | . | . |
| TS-08 | chr6 | 138200359 | 138200359 | C | T | TNFAIP3 | stopgain SNV | NM_001270507 | p.Q593X | . | . | . |
| TS-15 | chr6 | 138198305 | 138198305 | G | T | TNFAIP3 | stopgain SNV | NM_001270507 | p.E300X | . | . | . |
| TS-16 | chr6 | 138192527 | 138192528 | CA | - | TNFAIP3 | frameshift deletion | NM_001270507 | p.55_55del | . | . | . |
| TS-16 | chr6 | 138192655 | 138192655 | - | A | TNFAIP3 | frameshift insertion | NM_001270507 | p.T97fs | . | . | . |
| TS-07 | chr6 | 138201327 | 138201327 | A | T | TNFAIP3 | stopgain SNV | NM_001270507 | p.K676X | . | . | . |
| TS-17 | chr6 | 138196897 | 138196897 | C | - | TNFAIP3 | frameshift deletion | NM_001270507 | p.Q187fs | . | . | . |
| TS-18 | chr6 | 138192563 | 138192563 | - | C | TNFAIP3 | frameshift insertion | NM_001270507 | p.A67fs | . | . | . |
| TS-18 | chr6 | 138196897 | 138196897 | C | - | TNFAIP3 | frameshift deletion | NM_001270507 | p.Q187fs | . | . | . |
| TS-35 | chr16 | 2130324 | 2130324 | T | G | TSC2 | missense SNV | NM_001077183 | p.Y1142D | D | D | D |
| TS-06 | chr16 | 2134418 | 2134418 | G | C | TSC2 | missense SNV | NM_001077183 | p.G1332R | T | P | N |
| TS-37 | chr16 | 700281 | 700281 | T | C | WDR90 | missense SNV | NM_145294 | p.S103P | D | D | D |
| TS-18 | chr16 | 716010 | 716016 | GCGGCTG | - | WDR90 | frameshift deletion | NM_145294 | p.1499_1501del | . | . | . |

**Supplementary Table 6-1. Clonality analysis**

| Sample | Chr | Start | End | Ref | Alt | Gene | Alt^1^ | Coverage^2^ | VAF^3^ | Dominant clone's central VAF | clonal | Sub  clonal |
| --- | --- | --- | --- | --- | --- | --- | --- | --- | --- | --- | --- | --- |
| WG-01 | 6 | 138200107 | 138200107 | - | C | TNFAIP3 | 25 | 59 | 0.423 | 0.241 | O | . |
| WG-02 | 6 | 138196087 | 138196088 | AC | - | TNFAIP3 | 13 | 64 | 0.203 | 0.27 | O | . |
| WG-03 | 6 | 138199933 | 138199933 | G | T | TNFAIP3 | 13 | 27 | 0.481 | 0.303 | O | . |
| WG-08 | 3 | 176755901 | 176755901 | G | C | TBL1XR1 | 12 | 63 | 0.19 | 0.244 | O | . |
| WG-08 | 3 | 176768267 | 176768267 | C | T | TBL1XR1 | 12 | 59 | 0.203 | 0.244 | O | . |

**Supplementary Table 6-2. Aligned reads in A20 from Targeted-seq**

| Sample | Total reads | reads mapped on A20 | normalized value | Homozygous deletion |
| --- | --- | --- | --- | --- |
| TS-19 | 12647325 | 7950 | 0.00063 | O |
| TS-20 | 22771901 | 15858 | 0.0007 | O |
| TS-21 | 11907587 | 8901 | 0.00075 |  |
| TS-35 | 20544455 | 18236 | 0.00089 |  |
| TS-42 | 18042700 | 19277 | 0.00107 |  |
| TS-27 | 17204328 | 18518 | 0.00108 |  |
| TS-06 | 11916490 | 13090 | 0.0011 |  |
| TS-17 | 23454820 | 26304 | 0.00112 |  |
| TS-45 | 11781911 | 15335 | 0.0013 |  |
| TS-15 | 17441960 | 23604 | 0.00135 |  |
| TS-14 | 24190293 | 35822 | 0.00148 |  |
| TS-37 | 18680621 | 28646 | 0.00153 |  |
| TS-03 | 17037151 | 26165 | 0.00154 |  |
| TS-28 | 14408500 | 22356 | 0.00155 |  |
| TS-09 | 20537270 | 32215 | 0.00157 |  |
| TS-44 | 15611530 | 24913 | 0.0016 |  |
| TS-04 | 13610074 | 21743 | 0.0016 |  |
| TS-25 | 18277608 | 29273 | 0.0016 |  |
| TS-10 | 15484122 | 25598 | 0.00165 |  |
| TS-24 | 24584782 | 42138 | 0.00171 |  |
| TS-02 | 17761037 | 31365 | 0.00177 |  |
| TS-39 | 27668686 | 49491 | 0.00179 |  |
| TS-16 | 13627068 | 24475 | 0.0018 |  |
| TS-08 | 25491670 | 45823 | 0.0018 |  |
| TS-05 | 22815951 | 41134 | 0.0018 |  |
| TS-11 | 17198942 | 31181 | 0.00181 |  |
| TS-07 | 21566421 | 39642 | 0.00184 |  |
| TS-26 | 13712490 | 25312 | 0.00185 |  |
| TS-43 | 28745254 | 53117 | 0.00185 |  |
| TS-01 | 15674594 | 29524 | 0.00188 |  |
| TS-13 | 20700719 | 39296 | 0.0019 |  |
| TS-33 | 22473320 | 43633 | 0.00194 |  |
| TS-22 | 20449372 | 40262 | 0.00197 |  |
| TS-23 | 18302029 | 36145 | 0.00197 |  |
| TS-18 | 11227278 | 24345 | 0.00217 |  |
| TS-38 | 21536754 | 49742 | 0.00231 |  |
| TS-36 | 29258775 | 70890 | 0.00242 |  |
| TS-29 | 20668048 | 53629 | 0.00259 |  |
| Average | | | 0.001612632 |  |
| Standard deviation | | | 0.000454632 |  |
